# How Perceived Crowding Shapes Tourists’ Impulsive Buying in Internet-Famous Supermarkets: The Roles of Bipolar Emotions and Brand Trust

**DOI:** 10.3390/bs16091710

**Published:** 2026-09-21

**Authors:** Yayan Lu, Xiaoyan Zhang, Yizhuo Xiao, Di Liu, Jianchao Xi

**Affiliations:** 1 School of Geography and Tourism, Henan Normal University, Xinxiang 453000, China; luyayan16@mails.ucas.ac.cn (Y.L.); 1000608605@smail.shnu.edu.cn (Y.X.); 2022136@htu.edu.cn (D.L.); 2 Key Laboratory of Regional Sustainable Development Modeling, Institute of Geographic Sciences and Natural Resources Research, Chinese Academy of Sciences, Beijing 100101, China; 3 Institute of Business Administration, University of Perpignan via Domitia, 66860 Perpignan, France; xiaoyan.zhang@etudiant.univ-perp.fr; 4 International Institute of Science and Engineering of Perpignan, Henan Normal University, Xinxiang 453000, China

**Keywords:** internet-famous supermarkets, perceived crowding, emotion, impulsive buying, brand trust, SOR theory

## Abstract

This study investigates the impact mechanism of perceived crowding on consumer impulsive buying in the context of internet-famous supermarkets. Based on environmental psychology, this study proposes a moderated mediation model. Survey data from 565 tourists, collected between December 2024 and January 2026, were analyzed using structural equation modeling with SPSS 27.0, AMOS 28.0, and PROCESS macro 4.1. The results reveal that perceived social and spatial crowding exert divergent effects on impulsive buying and positive and negative emotions. Positive emotions act as a significant mediator between spatial crowding and impulsive buying, whereas social crowding drives impulsive buying primarily through direct social contagion rather than emotional pathways. Furthermore, while brand trust was hypothesized to broadly moderate this process, results reveal a limited and counterintuitive moderating role. It significantly interacts only with perceived social crowding, unexpectedly dampening its positive effects on both impulsive buying and positive emotions, whereas all other moderation and moderated mediation hypotheses remain unsupported. This research clarifies the distinct emotional mechanisms of perceived crowding and the specific suppressing role of brand trust, enriching environmental psychology and cognitive appraisal theory while providing practical guidance for the sustainable development of internet-famous retail.

## 1. Introduction

In the context of deeply integrated global tourism development, the convergence of social media and retail has given rise to internet-famous supermarkets (known in China as the *wanghong shangchao*), retail establishments that attract massive visitor flows not merely through product offerings but through viral online visibility and experiential appeal (C. Shen & Cheng, 2025; X. Yang et al., 2025). Unlike conventional retail environments, these supermarkets function as hybrid spaces where shopping and tourism converge, as consumers travel considerable distances to visit them, treat the shopping trip as a tourism experience, and engage in consumption behaviors shaped by both the physical environment and the digital narratives that drew them there (L. Cao, 2025). This phenomenon represents a paradigm shift in consumer spatial behavior, where the boundary between retail and tourism becomes increasingly blurred (Hu et al., 2026; C. Shen & Cheng, 2025). Moreover, unlike traditional shopping tourism centered on duty-free luxury consumption or souvenir purchasing, internet-famous supermarkets draw tourists primarily through experiential appeal, namely superior service quality, unique product offerings, and the social capital of checking in at trending locations (Y. Xie et al., 2026). In China, this trend has accelerated dramatically, with retail destinations such as Pang Donglai in Xuchang, Sam’s Club in multiple cities, and Hema Fresh stores becoming must-visit attractions for tourists, while social media platforms (e.g., Xiaohongshu, Douyin) serve as the primary channels through which these venues gain visibility and attract visitor flows (Zheng et al., 2026). The scale of this phenomenon is substantial, as evidenced by the 2024 National Day holiday when Pang Donglai’s Xuchang stores received over 3 million visitor trips, with approximately 85% identified as tourists^1^. This pattern, where a conventional supermarket becomes a regional tourism anchor, exemplifies the unique dynamics of internet-famous retail destinations.

However, internet-famous supermarkets face a distinctive paradox in that the very social media visibility that drives tourist inflows simultaneously creates extreme crowding conditions that may undermine the consumer experience. Unlike conventional retail environments where crowding is primarily a function of store layout and local demand, internet-famous supermarkets experience crowding that is socially amplified, driven by viral content, seasonal tourism patterns, and the fear of missing out psychology that compels visitors to converge on trending locations (Hu et al., 2026). Pang Donglai serves as an illustrative case, as despite being celebrated as a benchmark brand in China’s retail industry for its exceptional service and deep customer trust, its physical stores routinely operate at or beyond capacity, with peak-hour crowd density reaching levels that significantly constrain shoppers’ physical movement and psychological comfort. Critically, this crowding phenomenon is not unique to Pang Donglai but characterizes the broader category of internet-famous supermarkets, where any retail venue whose social media-driven popularity transforms it into a tourism magnet faces similar crowding pressures (Gretzel, 2019; Song & Wondirad, 2023). As a common and critical issue in such hybrid retail-tourism environments, crowding perception, comprising both spatial crowding (the perceived constraint of limited physical space) and social crowding (the perceived density of human presence), jointly influences tourists’ emotional states (Blut & Iyer, 2020; Zhang et al., 2018), which in turn may shape their impulsive buying through distinct psychological pathways (Sohn & Lee, 2017).

Existing research has established a foundational understanding of the relationships between crowding perception, emotions, and impulsive buying. Perceived spatial crowding has been shown to be significantly positively correlated with negative emotions such as anxiety and discomfort (Zhang et al., 2018), whereas moderate social crowding may elicit positive emotions by creating a lively, energizing atmosphere (Knoeferle et al., 2017; Van Rompay et al., 2012). The mediating role of emotion in the crowding-buying link has been documented through two broad pathways. Positive emotions encourage impulsive buying by reducing rational constraints and enhancing hedonic engagement (Casado-Aranda et al., 2022a; Lerner et al., 2015), whereas negative emotions may either inhibit or paradoxically promote impulsive buying through stress-coping and compensatory consumption mechanisms (Durante & Laran, 2016; Ferreira et al., 2017; Park et al., 2022).

Despite its merits and contributions to perceived crowding on impulsive buying, two critical theoretical gaps remain. First, prior studies have predominantly treated emotion as a unidimensional or single-valenced mediator, failing to simultaneously evaluate the differential effects of spatial and social crowding on both positive and negative emotions within a single integrative framework. This limitation is particularly consequential in light of the bipolar nature of emotional experience: positive and negative emotions are not mutually exclusive endpoints on a single continuum but can coexist within the same individual (Cacioppo & Berntson, 1994; Larsen et al., 2001). In crowded retail settings, consumers may simultaneously experience excitement from the vibrant social atmosphere and frustration from spatial constraints, creating an approach-avoidance tension that existing unidirectional emotion models fail to capture (Manthiou et al., 2020). The failure to account for this bipolar emotional complexity limits our understanding of how crowding ultimately shapes impulsive buying, as positive and negative emotions may exert opposing influences on the same behavioral outcome through parallel pathways (D. Cao et al., 2024). Although the differential effects of spatial and social crowding on emotions have been acknowledged (Apsari & Wibawa, 2026; Bandyopadhyay, 2020), the specific pathways through which each dimension of crowding triggers distinct bipolar emotional responses, and how these emotions independently mediate the relationship to impulsive buying, remain underexplored. Second, and equally important, the boundary conditions that shape how consumers respond to crowding remain theoretically underdeveloped. Brand trust, defined as a consumer’s confident expectation that a brand will deliver on its promises and act in the consumer’s interest (Chaudhuri & Holbrook, 2001) may function as a critical psychological resource that determines whether consumers appraise crowding as an opportunity or a threat. In high-trust retail environments, consumers may exhibit greater tolerance for crowding, perceiving it as a signal of popularity rather than a threat to service quality (X. Huang et al., 2018). Conversely, in low-trust environments, the same level of crowding may be interpreted as evidence of poor management, amplifying negative emotional responses. This trust-based attributional asymmetry suggests that brand trust fundamentally alters the crowding-emotion-behavior cascade by attenuating the negative emotional impact of crowding while amplifying positive emotional pathways (Delgado-Ballester & Luis Munuera-Alemán, 2001; Mehta et al., 2013). Without accounting for brand trust, the generalizability of crowding-emotion-behavior findings across different retail contexts remains uncertain, a limitation that prior research has not adequately addressed.

Internet-famous supermarkets, characterized by high consumer traffic driven by destination appeal and strong brand trust, provide a theoretically meaningful context for addressing these gaps. In such settings, the concurrence of positive and negative emotional responses to crowding is especially likely, as the excitement of visiting a trending destination coexists with the discomfort of physical constraint. Moreover, the prominent role of brand trust in these environments allows for a direct examination of how trust shapes the appraisal of crowding and, consequently, the emotional and behavioral pathways that follow. Against this backdrop, the present study attempts to enrich the knowledge of how perceived crowding shapes impulsive buying by addressing the following research questions: First, how do spatial and social crowding differentially influence consumers’ positive and negative emotions? Second, through what specific parallel mediating pathways do bipolar emotions transmit the effects of crowding perceptions to impulsive buying? Third, does brand trust moderate the relationships between (a) perceived crowding and emotions and (b) emotions and impulsive buying, and if so, to what extent?

To cope with these challenges, we present a comprehensive framework comprising a stimulus–organism–response (SOR) model that positions crowding perception as the stimulus, emotion as the internal state, and impulsive buying as the response, with brand trust as a key moderator. The study sets out to (1) reveal the distinct emotional mediation mechanisms linking spatial/social crowding to impulsive buying, (2) verify brand trust’s moderating role in the two-stage pathway, and (3) offer theoretical insights for high-trust retail enterprises to enhance their environmental strategies and consumer guidance.

## 2. Literature Review and Hypotheses

### 2.1. SOR Theory

SOR theory is a classic theoretical model for explaining the relationship between environmental stimuli and behavioral decisions. Its core logic posits that external stimuli (S) induce changes in psychological states (O) which subsequently lead to behavioral responses (R), emphasizing the mediating role of psychological states (Mehrabian & Russell, 1974). This theory has been widely integrated into tourism and marketing literature as a well-established framework for deciphering how diverse external factors elicit specific behaviors through individual emotional or cognitive variables. For example, M. J. Kim et al. (2020) revealed that realistic stimuli in virtual reality can evoke emotional responses such as pleasure and presence, thereby significantly enhancing tourists’ revisit intentions. Su and Swanson (2017) demonstrated that the stimulus of destination social responsibility requires the mediation of positive or negative consumption emotions and affective identification to cultivate environmentally responsible behavior. Furthermore, Gu et al. (2023) pointed out that stimuli including interactivity and information richness in live streaming significantly strengthen continuous participation intentions by elevating consumers’ perceived pleasure and sense of social support, particularly among younger demographics. Although existing literature has fully validated the universality of emotional variables within SOR theory, how perceived crowding acts as a core environmental stimulus to affect impulsive buying through the dual pathways of positive and negative emotions in the specific context of internet-famous supermarkets remains to be further clarified.

Therefore, this study adopts the SOR theory as an analytical framework to investigate tourists’ impulsive buying in internet-famous supermarkets. In particular, perceived crowding in the retail environment may influence tourists’ emotional responses. This environmental stimulus can affect the internal organism state of tourists, with both positive and negative emotions serving as important emotional responses. Ultimately, these emotional responses will influence tourists’ impulsive buying. Thus, this study explores the mechanisms through which perceived crowding shapes tourists’ impulsive buying based on the SOR theory, where perceived crowding (including spatial and social dimensions) is used as the external stimulus (S), emotion (including positive and negative emotions) is used as the internal organism response (O), and impulsive buying is used as the behavioral response (R).

### 2.2. Perceived Crowding and Impulsive Buying

Perceived crowding, a central concept in environmental psychology and consumer behavior, is defined as an individual’s subjective assessment of physical space limitations and social density in a given environment (Machleit et al., 1994; Stokols, 1972). Existing research commonly divides it into two dimensions, namely perceived spatial crowding and perceived social crowding. The former focuses on the physical constraints of the environment, such as movement restrictions caused by narrow aisles or densely arranged merchandise (Blut & Iyer, 2020; Eroglu et al., 2022). Conversely, the latter involves the psychological experience stemming from social interaction, which can include feelings of social stress or a perception of a lively atmosphere due to high-density crowds (Zhang et al., 2018).

Perceived spatial crowding links to tourists’ physical assessment of available space in a given environment. Under conditions of spatial constraint, individuals tend to experience an anxious sense of confinement, and such emotional responses further translate into specific consumption decisions (Blut & Iyer, 2020; J. Zhao et al., 2022). Despite evidence indicating that physical spatial constraints might suppress impulsive behavior (A. J. Xu & Albarracín, 2016), compensatory consumption theory provides a more generalizable theoretical lens for explaining why spatial crowding provokes impulsive buying. The core proposition of this theory holds that individuals tend to engage in consumption behavior to indirectly fulfill psychological needs deprived by environmental threats (R. Wang & Tian, 2025). Spatially crowded environments restrict tourists’ physical freedom, a deprivation that may elicit psychological reactance (Otterbring, 2016). In an effort to compensate for this diminished sense of control, consumers engage in variety-seeking behavior to reclaim their personal sovereignty. Levav and Zhu (2009) demonstrated that individuals exposed to spatial restriction exhibit a stronger tendency to select diverse product categories, as this reflects a typical defensive consumption behavior strategy that compensates for the lack of control through diversified choices. Meanwhile, the perceived spatial crowding stemming from an oppressive physical environment typically generates in individuals a strong sense of urgency (Blut & Iyer, 2020) and a pronounced desire to escape the environment (DeVellis & Thorpe, 2022). To promptly alleviate the unpleasant sensory experience and accomplish their shopping purposes, tourists are inclined to expedite their purchasing choices. This strategy substantially compresses the processes of information search and evaluation of alternatives and, more importantly, reflects consumers’ tendency toward cognitive simplification under dual spatiotemporal pressures. Consequently, individual purchase decisions frequently depart from established plans and eventually result in impulsive buying (Iyer et al., 2020; X. S. Liu et al., 2022).

In settings characterized by high human density, the concentration of crowds typically functions as a social proof signal, eliciting a heuristic belief among consumers that a greater number of people signifies better quality. This cognitive bias implicitly magnifies the allure and favorable evaluation of the retail context. When exposed to high-density social surroundings, consumers typically decode crowding as an external proxy for high popularity or substantial product value. Consequently, their behavioral motivation reverses from avoidance to approach, and this psychological conversion directly supplies the affective basis for impulsive buying (Blut & Iyer, 2020; Thomas & Saenger, 2020). The underlying mechanism by which perceived crowd density drives impulsive buying finds a systematic explanation within the social contagion theory. This theory holds that consumers’ keen observation of others’ choices and purchase behaviors generates a distinct social stimulus in crowded consumption environments. This exposure prompts a behavioral assimilation tendency within the observer, thereby precipitating unplanned purchasing or impulsive shopping (J.-G. T. Li et al., 2009). Within internet-famous retail environments, the prevalent phenomenon of rush buying from surrounding consumers readily triggers tourists’ herd mentality. Accordingly, individuals tend to eschew intricate cost–benefit evaluations, opting instead to rely on collective behavior as a heuristic cue for their purchasing decisions. Knoeferle et al. (2017) revealed that a complex correlation exists between social density and purchasing behaviors. Rather than inhibiting consumption, a moderate degree of social density paradoxically boosts overall retail sales via a mutually contagious herding effect. Notably, the theoretical mechanisms underlying the effects of spatial and social crowding on impulsive buying are fundamentally distinct. Spatial crowding operates through an avoidance-based compensatory mechanism, wherein consumers seek to restore deprived autonomy and expedite decisions to escape unpleasant environments. In contrast, social crowding operates through an approach-based social contagion mechanism, wherein consumers interpret crowd density as a positive quality signal and assimilate others’ purchasing behaviors. This divergence indicates that the same behavioral outcome, namely impulsive buying, may arise from diametrically opposed motivational origins depending on the type of crowding perceived. Prior studies have largely examined these two mechanisms in isolation rather than as complementary components of a unified framework. Based on the above analysis, the following hypotheses are proposed:

**Hypothesis** **1a.** *Perceived spatial crowding has a significant positive effect on impulsive buying.*

**Hypothesis** **1b.** *Perceived social crowding has a significant positive effect on impulsive buying.*

### 2.3. Perceived Crowding and Emotion

The emotional consequences of perceived crowding manifest through divergent pathways depending on its specific dimensions. Notably, the adverse impact of perceived spatial crowding on emotion has been extensively substantiated (Blut & Iyer, 2020). This effect is particularly pronounced in high-density retail environments, where densely arranged supermarket shelves can trigger environmental stress responses, thereby compromising the hedonic experience of consumers and lowering their sense of pleasure (Mantratzis et al., 2023). In contrast, the emotional influence of perceived social crowding demonstrates a pronounced contextual dependency. Within hedonic contexts, such as ancient village tourism or theme parks, moderate social crowding can foster a sense of social presence, which in turn amplifies positive affective responses like excitement and delight. For example, tourists with place attachment interpret the dense crowds in Xidi and Hongcun as cues of cultural vibrancy, which subsequently heightens their positive emotions (Zhang et al., 2018). However, in utilitarian contexts like convenience stores, excessive social crowding exacerbates negative emotions due to increased interpersonal friction (Byun & Mann, 2011). This divergence suggests that the two dimensions of crowding differ not merely in magnitude but in the nature of their appraisal processes. Spatial crowding is consistently appraised as a threat to personal space and autonomy, yielding relatively uniform negative emotional outcomes. In contrast, social crowding is appraised through a social cognition lens, which can yield either positive or negative evaluations depending on whether the context is framed as hedonic or utilitarian (Blut & Iyer, 2020). This asymmetry in appraisal flexibility may explain a consistent pattern in meta-analytic evidence. Spatial crowding reliably produces negative effects on customer outcomes, whereas the effects of social crowding are more variable and context-dependent (W. Liu et al., 2020).

While existing literature has revealed the association between perceived crowding and emotions, several limitations persist. First, contextual constraints are evident. The majority of inquiries concentrate on conventional tourist sites or traditional retail environments, leaving a dearth of targeted investigations into internet-famous supermarkets characterized by extreme social media visibility and destination appeal. Second, there is an oversight regarding emotional complexity. Current studies primarily examine the unidimensional influence of perceived crowding on singular affective states, thereby neglecting the potential coexistence of positive and negative affect (Russell & Pratt, 1980). In an internet-famous supermarket context, for instance, tourists may simultaneously experience frustration (a negative emotion) triggered by limited spatial space and excitement (a positive emotion) arising from a lively atmosphere, yet existing models fail to capture this emotional duality. To address the aforementioned limitations, this study focuses on the internet-famous supermarket context, conceptualizing perceived crowding along two distinct dimensions, namely perceived spatial crowding and perceived social crowding, to systematically explore their influence on both positive and negative emotions. Drawing on established consensus, constrained physical space directly curtails visitors’ behavioral freedom of movement, thereby eliciting environmental stress reactions. This experience not only heightens negative affect but also attenuates positive affect by restricting opportunities for social interaction. Thus, the following hypotheses are proposed:

**Hypothesis** **2a.** *Perceived spatial crowding has a significant positive effect on negative emotions.*

**Hypothesis** **2b.** *Perceived spatial crowding has a significant negative effect on positive emotions.*

Internet-famous supermarkets are fundamentally hedonic consumption environments focused on pleasurable experiences. In this context, moderate social crowding is not a negative interference but may instead be interpreted by tourists as a positive social signal indicating that the destination is highly popular and trustworthy. This perception fosters a sense of social presence, which subsequently amplifies positive emotions, including arousal and delight (Baker & Wakefield, 2012; Das & Varshneya, 2017; Gogoi, 2017). This vibrant social atmosphere effectively mitigates and potentially inhibits underlying negative emotions. Therefore, the following hypotheses are proposed:

**Hypothesis** **2c.** *Perceived social crowding has a significant negative effect on negative emotions.*

**Hypothesis** **2d.** *Perceived social crowding has a significant positive effect on positive emotions.*

### 2.4. Emotion and Impulsive Buying

As a typical unplanned consumption behavior, impulsive buying emerges as a spontaneous response devoid of prior planning and propelled by profound affective experiences (Stern, 1962). Later research delineated its central features, namely spontaneity and emotion-driven decision-making (Rook, 1987). The underlying psychological mechanisms of this phenomenon, which involves a temporary reduction in cognitive control, are primarily rooted in the depletion of self-control resources and the conflict between affect and cognition (Iyer et al., 2020; H. Xu et al., 2020). Within conventional retail environments, impulsive buying is predominantly triggered by external environmental stimuli, including shelf displays and promotional atmospheres (Bellini et al., 2017). In contrast, within emerging consumption contexts such as livestream e-commerce and shopping tourism, emotional arousal has been identified as a critical intrinsic driver of impulsive buying (Sohn & Lee, 2017; X. Xu et al., 2020).

As a crucial psychological mediator, emotion plays a pivotal role in consumers’ impulsive buying, and its impact exhibits a distinct bidirectional nature. First, the facilitative effect of positive emotions. Positive emotional states, including pleasure and excitement, have been extensively validated to substantially facilitate impulsive buying (Das & Varshneya, 2017; Gogoi, 2017). Neuroscientific studies elucidate this underlying mechanism by demonstrating that positive emotion elevates an individual’s psychological arousal. This elevated state of arousal transiently attenuates the cognitive control and rational inhibition functions of the prefrontal cortex while stimulating the neural reward circuitry, ultimately rendering consumers more susceptible to yielding to instant buying urges (Casado-Aranda et al., 2022b). Empirically, relevant studies reveal that in scenic-themed shopping environments, ambient atmospheric cues and service encounters, along with background visual complexity in live commerce streams, positively affect impulsive buying by evoking consumers’ positive emotions (Tong et al., 2022). Although these two lines of evidence operate at different analytical levels, they converge on the same conclusion. Positive emotions facilitate impulsive buying by weakening cognitive control mechanisms. This weakening occurs either through neural reward circuitry activation or through environmental cue-driven affective responses. This cross-level convergence substantially strengthens the theoretical robustness of the link between positive emotions and impulsive buying. Second, negative emotions have a more complex effect. The impact of negative emotions, including stress, anxiety, and frustration, on impulsive buying is notably more complex, as existing research reveals two fundamentally contrasting pathways. On the one hand, negative emotions may inhibit impulsive buying. When consumers experience stress or anxiety, stress-induced serotonin deficiency can suppress purchasing behaviors through the depletion of cognitive resources (Lichters et al., 2016). For example, tourists may exhibit greater caution when making shopping decisions under tight itineraries and physical or mental exhaustion. On the other hand, negative emotions may concurrently facilitate impulsive buying through the mechanism of compensatory consumption. Consumers may engage in shopping to mitigate adverse emotional states and pursue instant gratification and hedonic pleasure, thus accomplishing emotion regulation (Rook, 1987). These contrasting pathways raise a critical theoretical question: under what conditions does each mechanism prevail? Previous literature suggests that the inhibitory pathway (reducing willingness to buy) typically dominates in utilitarian or routine shopping contexts, where negative emotions signal task failure and simply deplete cognitive resources (Lichters et al., 2016). Conversely, the facilitative pathway (encouraging consumption via compensation) is more likely to activate in hedonic or tourism contexts, where individuals use consumption as an active coping mechanism to repair their mood and salvage the hedonic experience (Durante & Laran, 2016). Applying this theoretical boundary to internet-famous supermarkets, which are fundamentally hedonic tourism destinations, this study argues that the compensatory mechanism should theoretically prevail. Because non-local tourists absorb considerable sunk costs, they are highly motivated to offset travel-related stressors and emotional discomfort by acquiring psychological benefits through purchasing. Therefore, we hypothesize that within this specific tourism-retail paradigm, negative emotions will trigger a compensatory drive rather than a withdrawal response.

Overall, while existing literature provides foundational insights into the mechanisms whereby positive and negative emotions exert an influence on impulsive buying, there is a paucity of in-depth and comprehensive research on how these two types of emotions shape tourists’ impulsive buying in the unique context of internet-famous supermarkets. In particular, the literature has yet to systematically examine how the co-occurrence of positive and negative emotions, rather than either alone, jointly shapes impulsive buying. This question is especially pertinent in crowded internet-famous supermarket environments, where bipolar emotional experiences are likely. Consequently, this study presents the following research hypotheses:

**Hypothesis** **3a.** *Tourists’ negative emotions have a significant positive effect on impulsive buying.*

**Hypothesis** **3b.** *Tourists’ positive emotions have a significant positive effect on impulsive buying.*

### 2.5. The Mediating Role of Emotion

Emotion serves as a crucial psychological mediator linking environmental perception to consumer decision-making (S. Yang et al., 2022). Existing literature confirms that the emotional experience elicited by environmental cues constitutes the core mechanism driving consumers’ unplanned purchasing decisions (Lo et al., 2022). Recent scholarly advancements further underscore the necessity of examining emotion as a multidimensional mediator. Additional studies across emerging retail environments have consistently validated that positive and negative emotions serve as parallel yet distinct transmission mechanisms between environmental stimuli and consumer behaviors. For instance, recent research utilizing the SOR framework has demonstrated that while positive affective states consistently mediate the relationship between atmospheric facilitators and impulsive buying, negative affective states simultaneously mediate the pathway from environmental stressors to avoidance or compensatory behaviors (Iyer et al., 2020; Mandel et al., 2017). These findings reinforce the premise that treating emotion as a unidimensional construct obscures the true complex impact of retail environments. Consequently, it can be inferred that within the context of internet-famous supermarkets, tourists’ perceived crowding will initially trigger emotional responses, which subsequently influence their impulsive buying. However, as a prominent dimension of environmental perception in internet-famous supermarkets, the dual-pathway mechanism through which perceived crowding affects impulsive buying via both positive and negative emotions warrants further in-depth investigation.

On the one hand, the oppressive nature of the physical environment induces negative emotions in consumers, prompting them to engage in compensatory purchasing to alleviate this adverse psychological state (Rook, 1987). On the other hand, spatial crowding suppresses the generation of positive emotions, thereby indirectly diminishing purchase intentions by depriving consumers of pleasurable experiences (Mehrabian & Russell, 1974). These two pathways operate through opposing psychological logics. The negative emotion pathway reflects a reactive compensation mechanism, whereby consumers purchase to alleviate discomfort. In contrast, the positive emotion pathway reflects a hedonic facilitation mechanism, whereby consumers purchase because pleasure enhances approach motivation. Despite their divergent origins, these two pathways exert competing forces on impulsive buying. While the negative emotion pathway theoretically stimulates purchasing through compensatory motives, the suppressed positive emotion pathway simultaneously inhibits purchasing by removing hedonic drivers. This structural tension implies that spatial crowding activates a psychological tug of war, pulling consumer behavior in opposite directions and potentially canceling out the overarching main effect. Consequently, the following research hypotheses are proposed:

**Hypothesis** **4a.** *Perceived spatial crowding has a significant positive effect on impulsive buying through the mediation of negative emotions.*

**Hypothesis** **4b.** *Perceived spatial crowding has a significant negative effect on impulsive buying through the mediation of positive emotions.*

Moderate social crowding in hedonic scenarios can elicit positive emotions such as pleasure and excitement through social presence (Baker & Wakefield, 2012; Das & Varshneya, 2017; Gogoi, 2017). Similarly, in the context of internet-famous supermarkets, moderate crowd density can effectively enhance the sense of group participation. F. Li and Peng (2025) demonstrated that pleasure exerts a significant mediating effect in the process through which social presence drives willingness to pay. Furthermore, studies on live streaming shopping demonstrate that interactive stimuli boost continuous participation by enhancing the perceived pleasure of consumers (Y. Li & Peng, 2021). Accordingly, it can be argued that social cues released by moderate crowds can likewise strengthen the impulsive buying of tourists by stimulating positive emotional experiences. Comparing the mediation pathways of spatial and social crowding reveals a structural asymmetry. Spatial crowding primarily drives impulsive buying through the negative emotion pathway, a compensatory mechanism, while simultaneously suppressing the positive emotion pathway. Conversely, social crowding primarily drives impulsive buying through the positive emotion pathway, a hedonic facilitation mechanism, while simultaneously inhibiting the negative emotion pathway. This mirror-image pattern underscores the theoretical importance of examining both emotion valences rather than treating emotion as a unidimensional construct. Therefore, the following research hypotheses are proposed:

**Hypothesis** **4c.** *Perceived social crowding has a significant negative effect on impulsive buying through the mediation of negative emotions.*

**Hypothesis** **4d.** *Perceived social crowding has a significant positive effect on impulsive buying through the mediation of positive emotions.*

### 2.6. The Moderating Role of Brand Trust

Brand trust represents a composite psychological state that consumers cultivate through an assessment of brand reliability, performance competence, and emotional resonance. This trust is concretely expressed through confidence in the service quality and corporate values of the brand, as well as optimistic anticipations regarding enduring relational engagements (F. Li & Peng, 2025; Yao et al., 2013). In the context of tourism, brand trust emerges as the perceived reliability of experiences that visitors attribute to tourist destinations, tourism enterprises, or tourism products, exemplified by their endorsement of hotel service standards or their reliance on the scheduling provided by travel agencies (L. Xie et al., 2010).

Existing research on brand trust primarily focuses on its antecedent and outcome variables. These antecedent studies can be broadly categorized into two streams. The first is social-relational antecedents, which emphasize trust formation through interpersonal and social interaction processes. The second is informational-credibility antecedents, which emphasize trust formation through the perceived quality and transparency of information signals. In exploring antecedent variables within the social-relational stream, F. Li and Peng (2025) utilized the SOR theoretical framework to confirm that social presence ultimately exerted a significant positive impact on tourists’ willingness to pay a premium for digital cultural tourism products through the sequential mediation effects of pleasure and brand trust. Herein, brand trust acts as the pivotal variable bridging environmental stimuli and behavioral intentions. L. Xie et al. (2010) indicated that the brand citizenship behavior of tourism enterprise employees can directly enhance customer brand trust, and this effect is particularly pronounced in high-contact service environments. Similarly, Kamboj et al. (2018) found that user engagement in social media brand communities indirectly boosts brand loyalty through brand trust. Although these three studies converge on the centrality of social interaction in trust formation, they differ in their emphasis on the mode of social engagement. Within the informational-credibility stream, M. Kim and Kim (2020) pioneered the application of trust transfer theory to online tourism contexts and revealed the pivotal role of review authenticity in shaping traveler trust. Complementing this focus on information credibility, Laghari et al. (2025) found that in scenarios involving misinformation in influencer marketing, influencer type, attribution style, and brand response type significantly impact brand trust through the mediating mechanism of perceived brand responsibility. Extending the credibility logic to technological infrastructure, Zhu et al. (2024) further examined the capacity of blockchain technology to augment consumer trust in unfamiliar brands by advancing information transparency. Across these informational-credibility studies, a common thread emerges: brand trust is contingent upon the perceived credibility and transparency of information sources.

In the research on outcome variables, extensive research has substantiated the positive effects of brand trust. These outcome studies collectively reveal two distinct functions of brand trust. One is a behavioral promotion function that drives approach-oriented consumer behaviors. The other is a cognitive buffering function that attenuates the impact of negative experiences or perceived risks. Türk and Mustafa (2025) demonstrated that brand trust has a positive influence on store visit intentions. Representing the behavioral promotion function, S. Huang et al. (2024) found that green marketing initiatives enhance users’ repurchase intentions by elevating brand trust, thereby underscoring the core role of trust in maintaining enduring consumption relationships in the hospitality sector. Representing the cognitive buffering function, Yao et al. (2013) pointed out that high brand trust can reduce tourists’ sensitivity to service failures and effectively buffer the impact of negative experiences on satisfaction, demonstrating the cognitive buffering function of trust. Similarly, Hasan et al. (2021) revealed in their research on artificial intelligence voice assistants that brand trust can attenuate consumers’ perception of technological risks, consequently fostering higher acceptance. Although both studies demonstrate the buffering capacity of brand trust, the nature of the buffered threat differs, indicating that brand trust serves as a versatile defensive resource across different threat domains.

While current literature has comprehensively elucidated the direct pathways of brand trust, its moderating mechanism within the nexus of complex environmental stimuli, emotional responses, and behavioral outcomes has not been thoroughly investigated. Notably, the two functions of brand trust identified above, namely behavioral promotion and cognitive buffering, have been studied exclusively as direct effects. This leaves open the question of whether and how they operate as moderating mechanisms that reshape the relationships between environmental stimuli, emotional responses, and consumer behaviors. In contexts of perceived spatial crowding, brand trust provides an underlying cognition of product quality assurance, compelling tourists to perceive the products as highly desirable despite spatial constraints. Regarding perceived social crowding, X. Huang et al. (2018) noted that this environmental factor can foster brand attachment among tourists under specific circumstances. When tourists have substantial prior trust in a brand, this pre-existing trust synergizes with the environmentally induced attachment, thereby increasing the propensity for impulsive buying. Thus, the following hypotheses are proposed:

**Hypothesis** **5a.** *Tourists’ brand trust significantly and positively moderates the relationship between perceived spatial crowding and impulsive buying.*

**Hypothesis** **5b.** *Tourists’ brand trust significantly and positively moderates the relationship between perceived social crowding and impulsive buying.*

Drawing upon cognitive appraisal theory, brand trust functions beyond merely moderating the behavioral translation of environmental stimuli. It acts as an essential emotion regulation resource that directly shapes emotion generation via processes like attributional shift. Literature in environmental psychology corroborates that brand trust operates as a cognitive buffer to modify personal cognitive appraisals of stressful settings (Mehrabian & Russell, 1974; Su et al., 2022), which subsequently mitigates the adverse emotional effects of physical oppression. The findings of Donovan and Rossiter (1982) offer further evidence that retail environments dictate consumer behavior through emotional mediation, with individual traits playing a significant moderating role between environmental stimuli and emotional responses. Within the crowding contexts inherent to internet-famous supermarkets, the moderating mechanism of brand trust diverges based on the specific crowding typology. Specifically, in situations characterized by spatial crowding, high brand trust can weaken the negative impact of crowding on emotions through attributional shift or positive expectation. For instance, tourists can tolerate physical environment deficiencies due to their trust in a brand’s service quality (F. Li & Peng, 2025). Conversely, under conditions of social crowding, brand trust amplifies the tendency of consumers to decode perceived crowding as an affirmative indicator of brand attractiveness or a highly sought-after experience. Pedeliento et al. (2020) observed that brand trust magnifies the influence of reference groups. This divergent moderating pattern, namely attenuation under spatial crowding versus amplification under social crowding, mirrors the cognitive buffering and behavioral promotion functions identified earlier. This suggests that brand trust’s moderating role is not uniform but contingent on the type of environmental stimulus encountered. Based on the above discussion, this study proposes the following hypotheses regarding the moderating role of brand trust in the relationship between perceived crowding and emotions:

**Hypothesis** **6a.** *Tourists’ brand trust significantly and negatively moderates the relationship between perceived spatial crowding and negative emotions.*

**Hypothesis** **6b.** *Tourists’ brand trust significantly and positively moderates the relationship between perceived spatial crowding and positive emotions.*

**Hypothesis** **6c.** *Tourists’ brand trust significantly and negatively moderates the relationship between perceived social crowding and negative emotions.*

**Hypothesis** **6d.** *Tourists’ brand trust significantly and positively moderates the relationship between perceived social crowding and positive emotions.*

Furthermore, brand trust also plays a moderating role in the pathway from emotional experience to behavioral response. When negative emotions trigger compensatory buying, brand trust amplifies this facilitative effect through an emotional enhancement mechanism. S. Zhao and Liu (2024) revealed that anxiety translates more seamlessly into compensatory consumption motivation in contexts characterized by high brand trust, with this phenomenon being particularly salient among tourists exhibiting strong emotional attachment to the brand. L. Xie et al. (2010) confirmed that brand trust bolsters affective commitment, encouraging consumers to treat the brand as a trustworthy target for emotional venting and thereby solidifying the behavioral rationale of using shopping to relieve anxiety. These indicated that brand trust strengthens the behavioral expression of negative emotions by providing a psychologically safe consumption context. Meanwhile, brand trust reinforces the positive impact of positive emotions on impulsive buying by heightening the reward anticipation linked to brand reliability (Harrigan et al., 2021).

**Hypothesis** **7a.** *Tourists’ brand trust positively and significantly moderates the relationship between negative emotions and impulsive buying.*

**Hypothesis** **7b.** *Tourists’ brand trust positively and significantly moderates the relationship between positive emotions and impulsive buying.*

Overall, brand trust strengthens the interconnections among perceived crowding, emotions, and impulsive buying. It serves to further reinforce the mediating role of emotions throughout the entire process. Therefore, this study constructs a moderated mediation model and proposes the following hypotheses:

**Hypothesis** **8a.** *Tourists’ brand trust negatively moderates the mediating effect of negative emotions between perceived spatial crowding and impulsive buying.*

**Hypothesis** **8b.** *Tourists’ brand trust positively moderates the mediating effect of positive emotions between perceived spatial crowding and impulsive buying.*

**Hypothesis** **8c.** *Tourists’ brand trust negatively moderates the mediating effect of negative emotions between perceived social crowding and impulsive buying.*

**Hypothesis** **8d.** *Tourists’ brand trust positively moderates the mediating effect of positive emotions between perceived social crowding and impulsive buying.*

**Figure 1:**
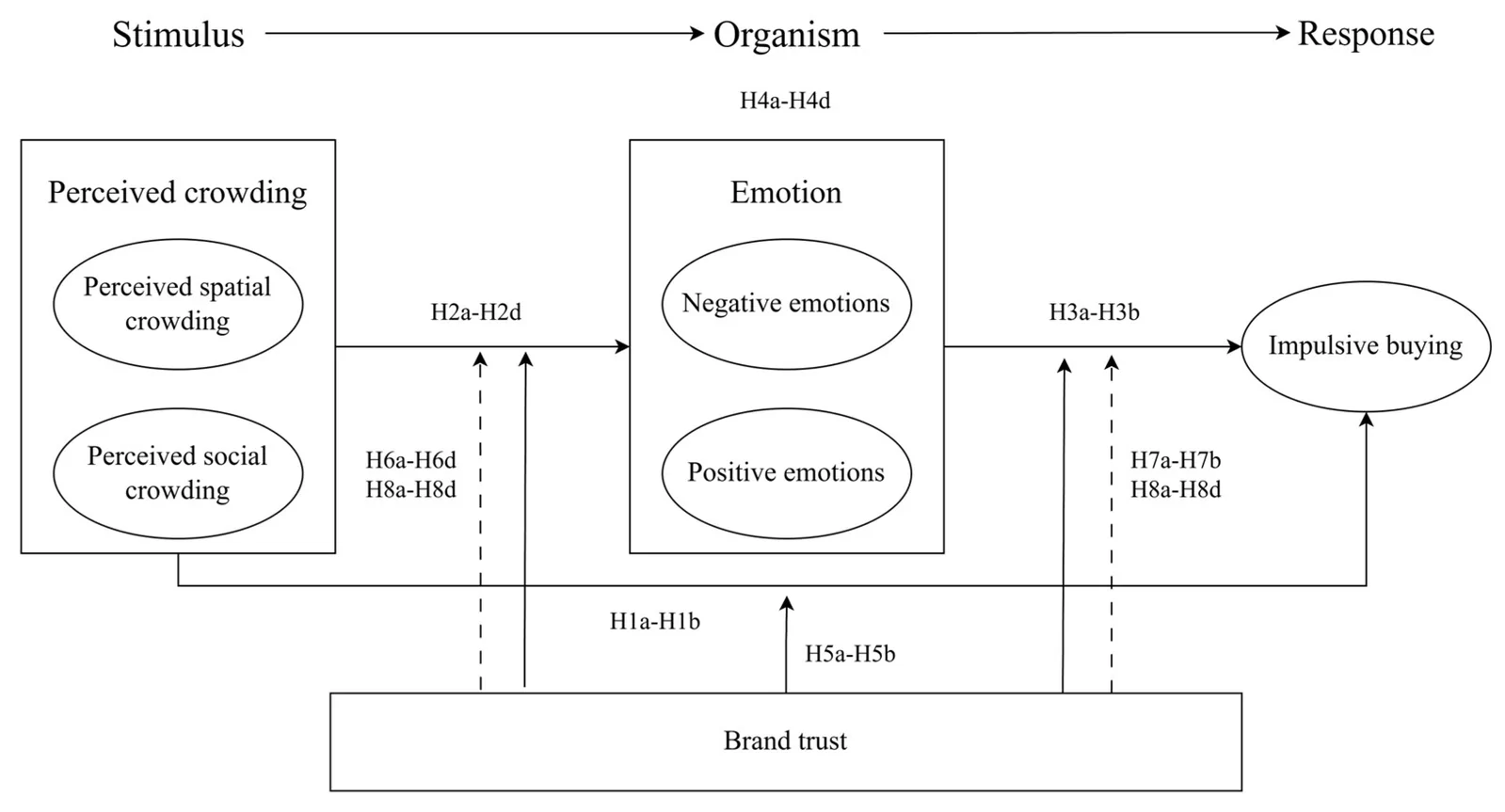
Conceptual model. Note: This model is grounded in the SOR framework. H4a–H4d denote the pathway of perceived crowding → emotions → impulsive buying; H5a–H5b represent the moderating pathways of brand trust in the perceived crowding → impulsive buying pathway; H6a–H6d indicate the moderating pathways of brand trust in the perceived crowding → emotions pathway; H7a–H7b signify the moderating pathways of brand trust in the emotions → impulsive buying pathway; the dotted lines refer to H8a–H8d, representing the moderated mediating pathways. Alt Text: A conceptual model diagram illustrating the hypothesized relationships among perceived crowding, emotion, impulsive buying, and brand trust. On the left, representing the “Stimulus”, the independent variable “Perceived crowding” includes two sub-dimensions: “Perceived spatial crowding” and “Perceived social crowding”. In the upper center, representing the “Organism”, the mediating variable “Emotion” contains two sub-dimensions: “Negative emotions” and “Positive emotions”. On the right, representing the “Response”, is the dependent variable “Impulsive buying”. At the bottom is the moderating variable “Brand trust”. The arrows indicate the following pathways: (1) a solid arrow from perceived crowding to impulsive buying represents the direct effect (H1a–H1b); (2) solid arrows from perceived crowding to emotion (H2a–H2d) and from emotion to impulsive buying (H3a–H3b) form the mediating pathway (denoted above as H4a–H4d); (3) upward solid arrows from brand trust represent moderating effects on the direct pathway (H5a–H5b), the first half of the mediation (H6a–H6d), and the second half of the mediation (H7a–H7b); (4) upward dotted arrows from brand trust to the mediating pathways denote the moderated mediating pathways (H8a–H8d).

In summary, the conceptual model of this study is illustrated in Figure 1.

## 3. Research Design

### 3.1. Study Site

**Figure 2:**
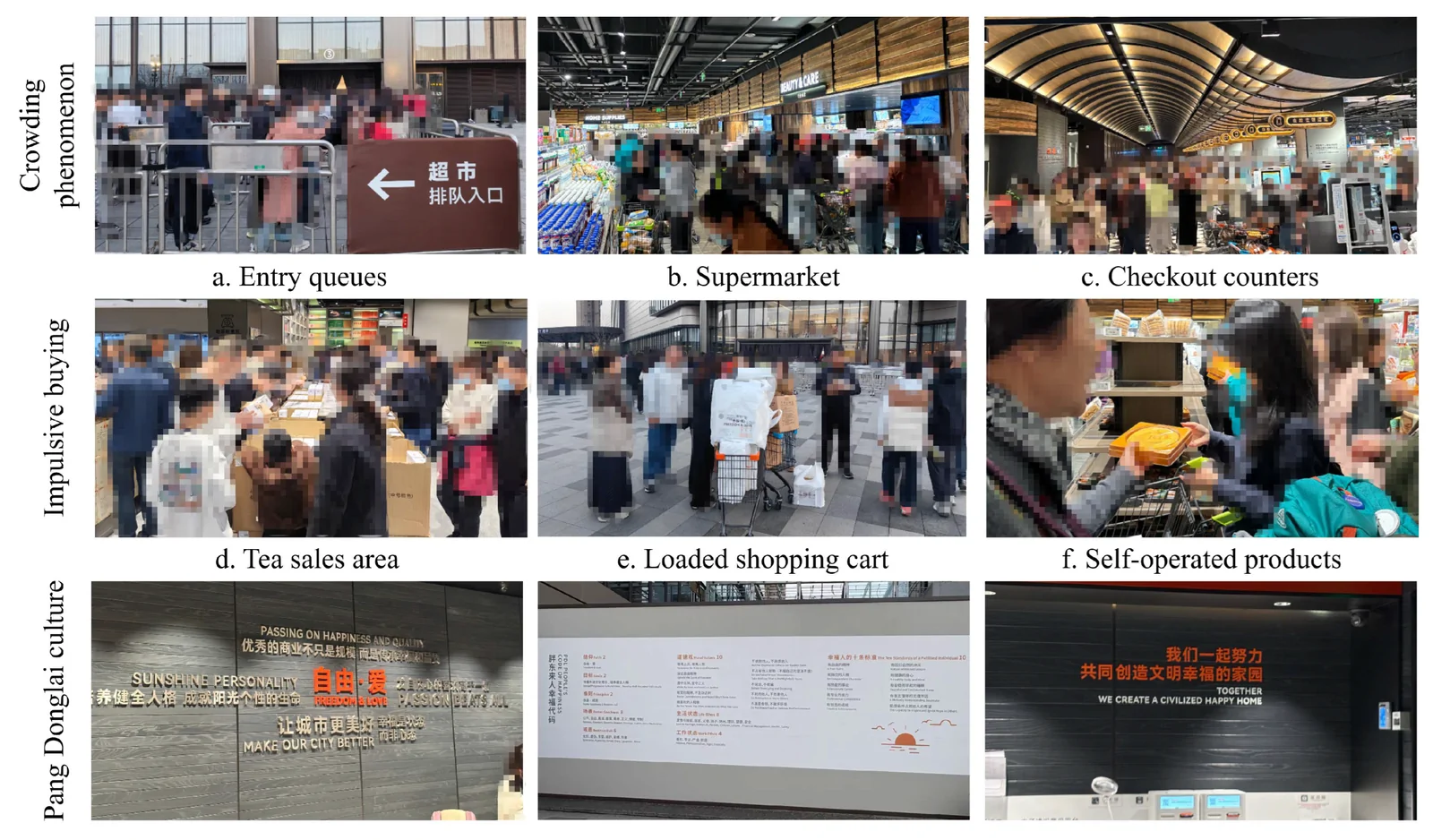
Study site.

This study selects Pang Donglai Commercial Group, located in Henan Province, China, as the case study site. Founded in 1995 with primary operations in the cities of Xuchang and Xinxiang, Pang Donglai has expanded into a multifaceted retail corporation encompassing supermarkets, department stores, dining, and entertainment facilities. Stimulated by social media dissemination, consumer word-of-mouth, and the emergence of destination-based consumption in recent years, Pang Donglai has transcended the conventional functional limits of retail venues. It has transformed into a paradigmatic internet-famous supermarket that integrates retail, experiential, and touristic appeal (Figure 2). On weekends and public holidays in particular, a large number of non-local visitors travel exclusively to Pang Donglai, driven primarily by motivations to shop, experience, check in at the destination, and sightsee. This phenomenon demonstrates pronounced features of tourist mobility and consumption agglomeration. Unlike standard retail outlets, Pang Donglai satisfies not only the functional need to purchase merchandise but also complex motivations, including engaging with a prominent commercial environment, absorbing local consumer culture, and facilitating social media sharing. Consequently, the retail environment at Pang Donglai extends beyond traditional routine shopping to display prominent internet-famous supermarket characteristics, thereby establishing an empirical basis for investigating visitor behavioral responses within specialized consumption spaces.

For a long time, Pang Donglai has maintained an operational philosophy centered on integrity and superior quality, thereby cultivating an exceptionally high degree of brand trust and consumer reliance. Through rigorous quality control, transparent profit margins, and exhaustive customer service, the enterprise attracts substantial volumes of non-local tourists. This immense popularity consequently subjects its retail locations to chronic hyper-saturated pedestrian traffic. Visitors frequently encounter severe spatial limitations, extended queues, and dense interpersonal proximity, particularly within high-demand merchandise sales zones, holiday peak periods, and concentrated display zones for viral products. Such extreme crowd density functions as potent external environmental stimuli while simultaneously generating significant perceptions of spatial crowding and social crowding within the limited retail space. Characterized by both spatial confinement and a collective celebratory atmosphere, this intricate crowding environment serves as an exceptionally fitting and representative experimental field. It enables this research to examine how varying dimensions of perceived crowding operate as external triggers that elicit psychological and behavioral reactions from consumers.

### 3.2. Questionnaire Design

Drawing upon a comprehensive literature review, this research synthesized established measurement scales. During October 2024, a panel of eight experts specializing in tourism management, tourism geography, marketing, and sociology was invited. These scholars, affiliated with the Institute of Geographic Sciences and Natural Resources Research of the Chinese Academy of Sciences, the Xinjiang Institute of Ecology and Geography of the Chinese Academy of Sciences, Xinjiang University, and Henan Normal University, evaluated and enhanced the structural integrity, item formulation, and phrasing of the survey instrument. Subsequently, a pilot study was conducted to correct identified issues and finalize the questionnaire. The questionnaire comprises seven sections assessing perceived spatial crowding, perceived social crowding, negative emotion, positive emotion, impulsive buying, brand trust, and basic demographic information. The measurements of both perceived spatial crowding and perceived social crowding were derived from the scales developed by (Machleit et al., 1994), with each comprising four items. It is important to note that these items were specifically selected and adapted to capture tourists’ subjective psychological evaluations of physical constraint and social density (e.g., feelings of restriction or perceived busyness), rather than objective physical metrics of crowd levels. This subjective operationalization aligns with the theoretical logic of our SOR framework. We posit that the effective environmental stimulus (S) triggering a consumer’s internal state is not the objective spatial density itself, but rather the individual’s psychological appraisal of that density. By measuring perceived crowding, we accurately capture the subjective stimulus that directly drives subsequent emotional (O) and behavioral (R) responses. The measurement of negative emotion was evaluated through a five-item scale adapted from the research of Chitturi et al. (2008) and Watson et al. (1988). The assessment of positive emotion was captured using four items based on Watson et al. (1988). The measurement of impulsive buying, incorporated three items, was operationalized using the scales formulated by Badgaiyan and Verma (2015). Drawing on the classical conceptualization of the “impulsive buying episode” (Rook, 1987), this construct was operationalized holistically to capture both the spontaneous psychological desire experienced during the shopping trip and the resulting behavioral outcome of unplanned purchasing. Thus, combining these indicators into a single latent construct accurately reflects the intensity of the actual impulsive buying behavior executed by tourists. The measurement of brand trust was quantified using a six-item scale proposed by B. Wang et al. (2022). To align with the specific context of internet-famous supermarkets, slight linguistic modifications were applied to the measurement items without altering their core semantic meaning. Responses for all items were recorded on a five-point Likert scale anchored at 1 (“strongly disagree”) and 5 (“strongly agree”).

### 3.3. Data Collection and Sample Profile

The target respondents for this study comprised tourists from regions outside the main urban area of Xinxiang City in Henan Province who had completed shopping experiences at Pang Donglai. To accurately identify the target population (i.e., non-local tourists) for this study, participants were required to answer three screening questions prior to completing the formal questionnaire, formulated in accordance with the World Tourism Organization’s definition of domestic visitors. First, they needed to confirm that their primary residence was located outside the main urban area of Xinxiang City (which allows for the inclusion of visitors from outer counties within the broader Xinxiang municipality). Second, they needed to confirm that their current trip involved a minimum travel duration of more than six hours. Third, they had to verify that their current itinerary included an actual shopping experience at Pang Donglai. Only respondents who met all these inclusion criteria were invited to participate in the formal survey.

Given that most tourists choose to visit Pang Donglai on weekends and public holidays, visitor traffic surges dramatically during these periods, creating the high-density conditions central to this study. To target the participant cohort who had experienced perceived crowding at Pang Donglai, data collection was conducted during weekends and public holidays. The data collection proceeded through three stages. In the first stage (2–4 October 2024), the research team conducted a pilot study at Pang Donglai in Xinxiang, yielding 72 valid responses. Through on-site observation and informal interviews with staff and visitors, the research team gained preliminary insights into visitors’ behavioral responses to crowded environments. Based on the pilot results, several issues were identified. First, the perceived crowding items did not adequately distinguish between spatial and social crowding dimensions. Second, the emotion items required greater specificity and alignment with established affect scales. Third, the impulsive buying items needed revision to better capture the construct in the retail context. The final questionnaire was completed by late October 2024. In the second stage, the formal survey was conducted from 20 December 2024 to 13 January 2025, yielding 384 valid responses. A supplementary survey was conducted from 13 to 18 January 2026, yielding 199 valid responses, because Pang Donglai had implemented a series of unprecedented crowd management measures, including real-name membership verification, purchase limits on popular items, temporarily shortened operating hours with entry cutoff times, on-site crowd flow regulation during holiday peaks, and the opening of a new Xinxiang store. These collectively reshaped customer flow distribution across all citywide stores, potentially altering tourists’ crowding perceptions. Furthermore, to ensure data integrity and mitigate common method bias, the questionnaire incorporated reverse-scored items, attention checks (e.g., “Please select strongly disagree”), and randomized ordering of response options. The survey sites were located in high-density areas within the retail environment, specifically the first floor and the third-floor supermarket of Pang Donglai. Ultimately, a total of 583 questionnaires were distributed. After excluding invalid questionnaires characterized by identical responses across all items or excessively short completion times, the study retained 565 valid questionnaires, resulting in an overall effective response rate of 96.9%. The descriptive statistical analysis of the sample characteristics is presented in Table 1.

## 4. Results

### 4.1. The Measurement Model

To ensure the reliability and stability of the measurement scales employed in this study, it is essential to assess data quality prior to conducting formal hypothesis testing. This study first examined the internal consistency reliability of each variable dimension by calculating Cronbach’s α coefficient. Following universally recognized methodological benchmarks (DeVellis & Thorpe, 2022), a Cronbach’s α coefficient exceeding 0.7 indicates an ideal level of scale reliability. As elucidated in Table 2, the reliability analysis outcomes demonstrate that the Cronbach’s α coefficients for all measurement dimensions significantly exceed the 0.7 cutoff criterion. This finding suggests that the measurement scales adopted in this study possess strong internal consistency and reliability, thereby confirming that the data quality is stable and trustworthy. Furthermore, this study conducted a confirmatory factor analysis (CFA) to evaluate the goodness of fit for the overall measurement model. The model exhibited strong performance across all critical fit indices. Specifically, the ratio of chi-square to degrees of freedom (χ^2^/dƒ) falls within the ideal range of 1 to 3. The root mean square error of approximation (RMSEA) remains within the acceptable threshold of less than 0.08. In addition, the results for the comparative fit index (CFI), incremental fit index (IFI), and Tucker–Lewis index (TLI) all reached excellent levels exceeding 0.9. The specific results are presented in Table 2. These metrics collectively provide compelling evidence that the proposed measurement model in this study aligns closely with the empirical sample data.

Accordingly, this study further examines the convergent validity, measured by average variance extracted (AVE) and composite reliability (CR), as well as the discriminant validity for each scale dimension. The standardized factor loadings of all measurement items on their designated dimensions are detailed in Table 2. With all standardized factor loadings exceeding 0.5, the items demonstrate strong representativeness of their associated latent variables. The AVE values for most constructs in this study exceed 0.5, and all CR values are above 0.7 (Hair et al., 2019). Although the AVE values for perceived spatial crowding (0.498) and perceived social crowding (0.460) are marginally below the 0.5 threshold, their CR values are well above 0.7. According to Fornell and Larcker (1981), the convergent validity of a construct is still deemed adequate if the CR is higher than 0.6, even if the AVE falls slightly below 0.5. Moreover, the discriminant validity evaluation highlights that the standardized correlation coefficients between any two given dimensions are consistently smaller than the square root of the AVE values (Table 3), and the heterotrait–monotrait (HTMT) values are all below 0.85 (Henseler et al., 2015) (Table 4). Therefore, this demonstrates that all the dimensions possess good discriminant validity. In summary, the findings substantiate that each dimension exhibits excellent convergent validity, composite reliability, and discriminant validity.

During the data preprocessing stage, descriptive statistics and normality tests were performed for all measurement items. The descriptive statistics reveal that the mean scores of all variables ranged between 2 and 5. The normality of each measurement item was evaluated using skewness and kurtosis metrics. According to the criteria proposed by Kline (2023), the data are assumed to be approximately normally distributed when the absolute value of the skewness coefficient is less than 3 and that of the kurtosis coefficient is less than 8. As illustrated in Table 5, the absolute values of both skewness and kurtosis coefficients of all measurement items in this study remain within these acceptable thresholds. Thus, the data for all measurement items satisfy the assumption of an approximate normal distribution.

### 4.2. Common Method Bias Testing

As the data for this research were collected via self-reported instruments, the potential for common method bias (CMB) exists. To proactively control for the sources of CMB, this study employed both procedural and statistical remedies. Procedurally, the questionnaire utilized well-established scales, ensured respondent anonymity, and randomized the order of items. The survey instructions underscored the anonymity and confidentiality of the respondents, clarifying that all collected data were strictly for academic research and that there were no right or wrong answers, encouraging participants to respond based on their actual experiences (Podsakoff et al., 2003). Additionally, reverse-scored items and attention checks were strategically embedded throughout the questionnaire to further reduce response pattern biases.

Statistically, Harman’s single-factor test was initially conducted to evaluate CMB. The unrotated exploratory analysis demonstrated that six factors possessed eigenvalues greater than 1, with the first extracted factor explaining 28.99% of the variance, which falls well below the critical 50% value (Fuller et al., 2016). Furthermore, to stringently evaluate common method bias beyond the traditional Harman’s test, an unmeasured latent method construct (ULMC) approach was utilized (Kock et al., 2021; Podsakoff et al., 2003). A latent common method construct was added to the measurement model and linked to all observed indicators. We then calculated the variance explained by this ULMC by squaring the standardized loadings of the paths connecting the ULMC to all observed items. The average variance extracted by the common method construct was 23.66%, which is substantially lower than the critical 50% threshold (Podsakoff et al., 2003). Consequently, both procedural controls and rigorous statistical assessments confirm that common method bias accounts for only a minor portion of the total variance and does not confound the substantive structural relationships of this research.

### 4.3. Structural Model

This study further employed structural equation modeling (SEM) to examine the influence relationships among the variables. The results indicate that χ^2^/dƒ = 2.534, RMSEA = 0.052, CFI = 0.962, IFI = 0.962, and TLI = 0.954, thereby demonstrating an excellent goodness-of-fit for the overall model. The findings reveal that perceived spatial crowding exerts a significantly negative influence on positive emotions (β = −0.491, *p* < 0.001), whereas it has a significantly positive impact on negative emotions (β = 0.675, *p* < 0.001). Conversely, perceived social crowding negatively and significantly impacts negative emotions (β = −0.134, *p* < 0.05), while it positively and significantly influences positive emotions (β = 0.138, *p* < 0.05). Furthermore, negative emotions impose a significant impact on impulsive buying (β = −0.157, *p* < 0.05), but in the opposite direction of the hypothesis. Positive emotions significantly and positively affect impulsive buying (β = 0.321, *p* < 0.001). While perceived spatial crowding fails to show a significant impact on impulsive buying (β = 0.039, *p* = 0.686), perceived social crowding exerts a significantly positive influence on impulsive buying (β = 0.161, *p* < 0.05). Consequently, hypotheses H1b, H2a to H2d, and H3b are supported, whereas hypotheses H1a and H3a are rejected. The specific test results are presented in Table 6.

**Figure 3:**
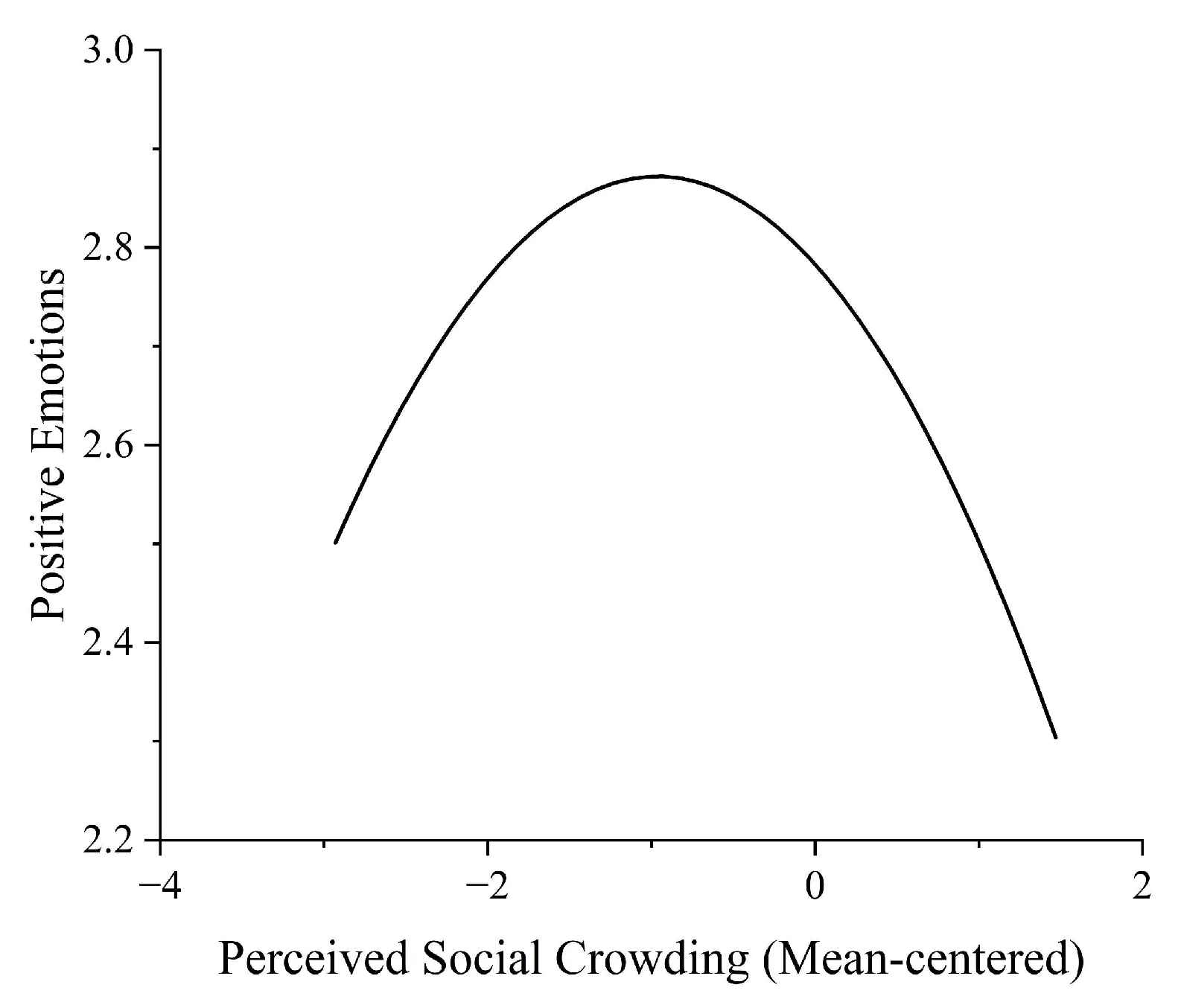
The inverted-U relationship between perceived social crowding and positive emotions. Alt Text: A line graph illustrating an inverted-U relationship between perceived social crowding and positive emotions. The horizontal axis represents mean-centered perceived social crowding, ranging from −4 to 2. The vertical axis represents positive emotions, ranging from 2.2 to 3.0. A single solid black curve starts at a lower value on the left, rises smoothly to reach a peak indicating maximum positive emotions at a moderate level of social crowding, and then slopes downward progressively as crowding increases toward the right. This demonstrates that positive emotions increase with initial crowding but decline significantly once crowding becomes excessive.

Furthermore, to address the theoretical possibility of a threshold effect in social crowding, we conducted a supplementary hierarchical regression analysis using SPSS 27.0 to test for potential nonlinear relationships. To ensure measurement accuracy, factor scores for perceived social crowding and emotions were extracted from the CFA measurement model using AMOS 28.0. The results revealed that while the quadratic term of perceived social crowding on impulsive buying was not significant (*p* = 0.073), it exerted a highly significant negative effect on positive emotions (β = −0.151, *t* = −3.500, *p* < 0.001, ΔR^2^ = 0.020). This indicates a significant inverted-U relationship (Figure 3), suggesting that while moderate social crowding enhances positive emotions, excessive crowding beyond a certain threshold significantly diminishes the hedonic experience.

### 4.4. Mediation Effect Analysis

This study employed SEM via AMOS 28.0 to compute user-defined estimands. The analysis utilized a bootstrapping procedure with 5000 resamples to generate 95% bias-corrected confidence intervals. The results indicate that the mediating effect of negative emotions between perceived spatial crowding and impulsive buying is not significant (β = −0.106, 95% CI = [−0.194, 0.040], *p* = 0.265), thus failing to support H4a. Conversely, positive emotions demonstrate a significant negative mediating effect between perceived spatial crowding and impulsive buying (β = −0.158, 95% CI = [−0.321, −0.078], *p* < 0.001), thereby supporting H4b. Furthermore, the mediating effects of negative emotions (β = 0.021, 95% CI = [−0.008, 0.168], *p* = 0.217) and positive emotions (β = 0.044, 95% CI = [−0.015, 0.229], *p* = 0.104) between perceived social crowding and impulsive buying are both not significant. Consequently, hypotheses H4c and H4d are rejected.

### 4.5. Moderation Effect Analysis

To rigorously evaluate the moderating effects, this study utilized Model 1 of the PROCESS macro for SPSS. To minimize the potential risk of multicollinearity, all continuous variables defining the interaction terms were mean-centered prior to model estimation. The statistical significance of the interaction terms and conditional effects was assessed using a bootstrapping procedure with 5000 resamples to yield 95% confidence intervals.

As presented in Table 7, most moderation hypotheses (H5a, H6a–H6c, H7a–H7b) are not supported. However, a noteworthy core finding is that the interaction terms for H5b and H6d achieved statistical significance, but in a direction opposite to the hypothesized direction. Specifically, rather than amplifying the stimulatory effect of perceived social crowding on impulsive buying, brand trust actually yields a suppressive effect. Similarly, brand trust fails to reinforce the positive influence of perceived social crowding on positive emotions as anticipated and instead demonstrates a significant negative impact.

**Figure 4:**
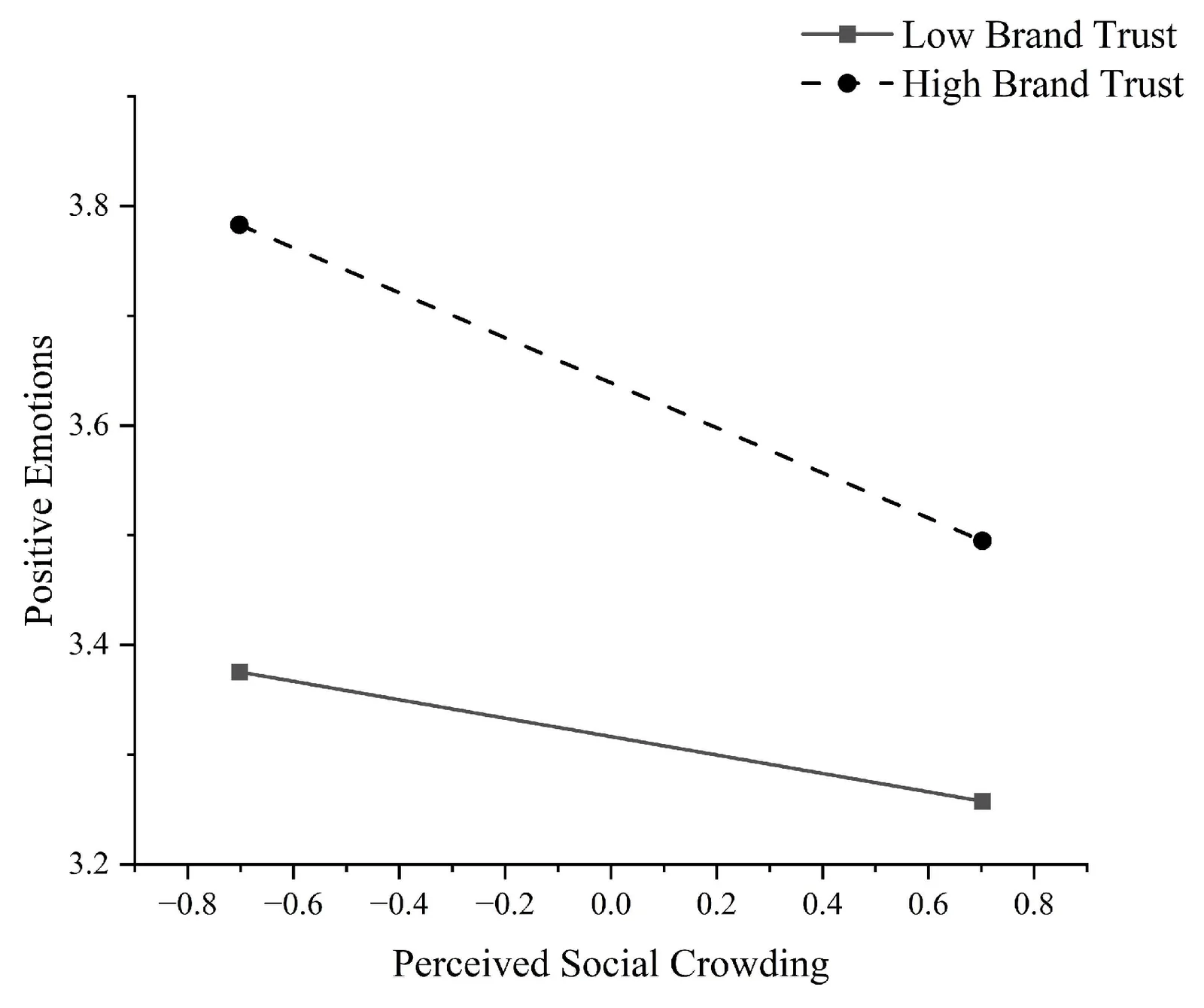
The moderating effect of brand trust on the relationship between perceived social crowding and positive emotions.

**Figure 5:**
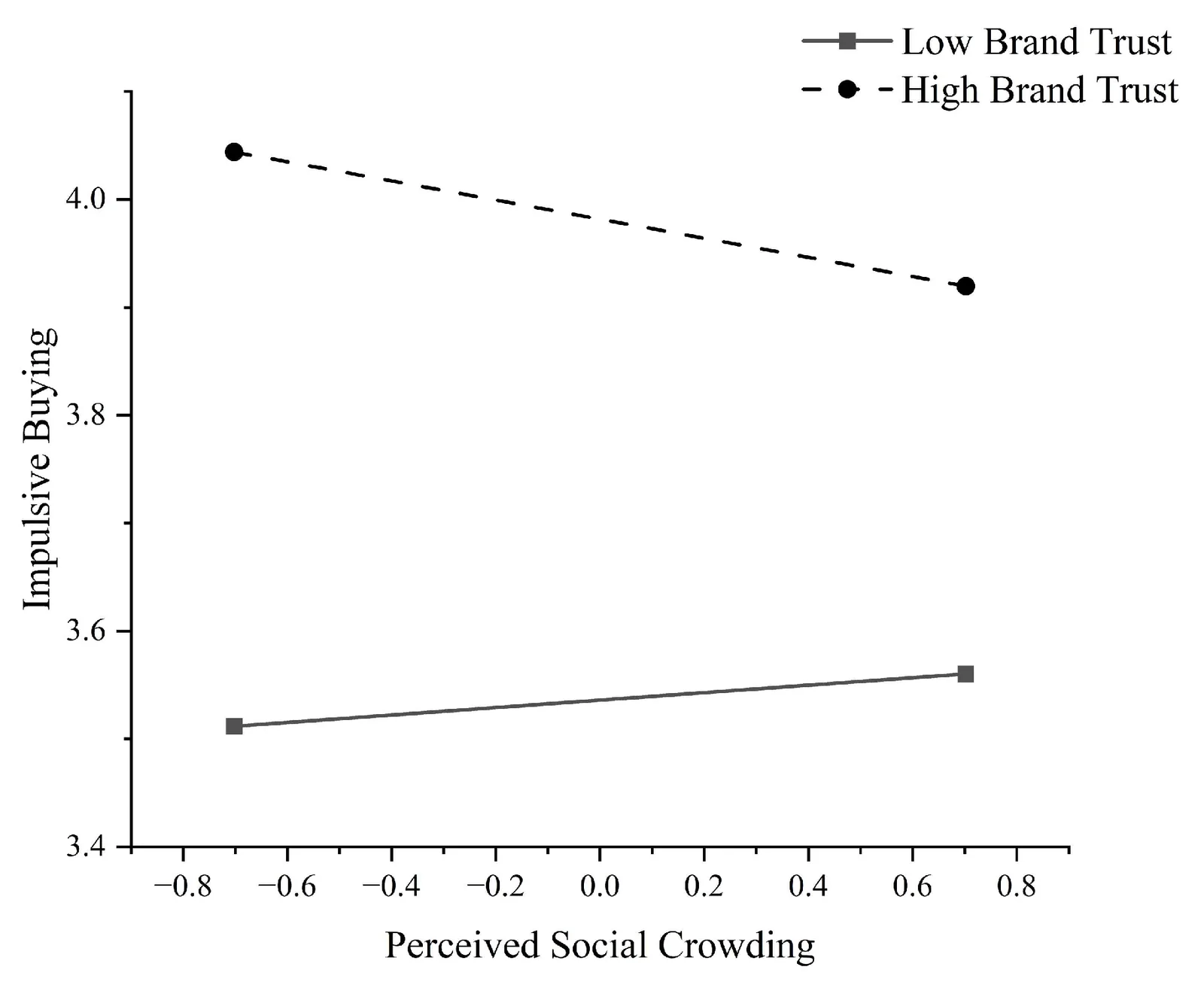
The moderating effect of brand trust on the relationship between perceived social crowding and impulsive buying.

To further elucidate the nature of these significant moderating effects, simple slope analyses were conducted, and interaction graphs were plotted at one standard deviation above and below the mean of brand trust. As illustrated in Figure 4, for tourists with high brand trust (+1 SD), perceived social crowding significantly reduces positive emotions (β = −0.205, *t* = −4.115, *p* < 0.001). Conversely, for tourists with low brand trust (−1 SD), this negative impact is less pronounced and non-significant (β = −0.084, *t* = −1.815, *p* = 0.070). Similarly, Figure 5 demonstrates that high brand trust unexpectedly dampens the positive effect of perceived social crowding on impulsive buying, exhibiting a marginally significant negative trend (β = −0.089, *t* = −1.808, *p* = 0.071), while the effect for those with low brand trust is non-significant (β = 0.035, *t* = 0.762, *p* = 0.446).

### 4.6. Moderated Mediation Effect Tests

To comprehensively evaluate the moderated mediation hypotheses, this study em-ployed Model 59 of the PROCESS macro. Consistent with the moderation analysis, all con-tinuous variables defining the interaction terms were mean-centered prior to model esti-mation. Because moderation occurs on both the first and second stages of the mediation process in Model 59, the indirect effect is a non-linear function of the moderator, meaning a single, formal “index of moderated mediation” cannot be computed mathematically. In-stead, following the guidelines of Hayes (2013), a difference analysis (i.e., probing the contrasts of conditional indirect effects) was applied. We explicitly contrasted the conditional indirect effects at high (+1 SD) and low (−1 SD) levels of the moderating variable (brand trust) using 5000 bootstrapped resamples to yield 95% confidence intervals. As shown in Table 8, the results indicate that in all four models, the differences in the indirect effects under different levels of the moderating variable (specifically high and low levels of brand trust) are not significant. Consequently, hypotheses H8a–H8d are not supported.

## 5. Conclusions and Discussion

### 5.1. Research Findings

This study focuses on internet-famous supermarkets to systematically reveal the complex mechanisms among tourists’ perceived crowding, emotion, brand trust, and impulsive buying in internet-famous supermarket contexts. The main conclusions derived from this research are presented below:

First, perceived social crowding exerts a significantly positive impact on impulsive buying, while the impact of perceived spatial crowding remains insignificant. This indicates that in internet-famous supermarket contexts, social crowding communicates the popularity value of internet-famous supermarkets. The scramble among tourists for tourism commodities, particularly viral products, catalyzes a bandwagon effect that subsequently precipitates impulsive buying. Diverging from prior research (Blut & Iyer, 2020), this investigation reveals that perceived spatial crowding does not serve as a significant driver of impulsive buying. This anomalous phenomenon can be plausibly explained by the distinctive operational characteristics of Pang Donglai, as corroborated by our qualitative interviews. First, the spatial layout and service infrastructure act as physical and psychological buffers. As noted by one visitor, the store maintains “sufficient internal passage space” and “clear directional signage” that prevent crowd density from translating into suffocating physical oppression (Interviewee E, see S4 in the Supplementary Materials). Second, the shopping context heavily dictates consumer mindset. For the massive influx of non-local visitors attracted by internet-famous supermarkets, traveling considerable distances implies they arrive with specific purchasing objectives. Under these high-involvement conditions, these consumers rely heavily on predetermined mental or physical shopping lists. Our interviews revealed that even when faced with dense crowds or frantic buyers, many non-local visitors adhere strictly to their planned items, such as tea or specific daily necessities, demonstrating a pronounced target-oriented rationality that inhibits spatial crowding from triggering spontaneous, unplanned purchases (Interviewee A and B, see S4 in the Supplementary Materials).

Second, perceived spatial crowding imposes a significant positive influence on negative emotions and a significantly negative effect on positive emotions. Conversely, perceived social crowding significantly and negatively impacts negative emotions while positively influencing positive emotions. When tourists perceive physical space restrictions, tourists experience an infringement upon their personal boundaries and a loss of behavioral freedom. Such physical discomfort and the frustration associated with hindered goals inherently provoke negative emotional states, including agitation and apprehension. This subsequently attenuates their enjoyment and favorable experiences throughout the tourism shopping endeavor, corroborating the conclusions drawn by Tran (2020). Within the internet-famous supermarket environment, especially during visits to prominent retail destinations such as Pang Donglai, dense pedestrian traffic is frequently construed as a remarkable indicator of exceptional brand appeal and superior merchandise quality. This vibrant retail climate satisfies the hedonic drives of consumers pursuing novelty and shared festivity while simultaneously fostering group identification (Cialdini, 2014), which in turn arouses positive affective responses like enthusiasm and expectancy. However, our supplementary analysis reveals that this positive influence does not follow a simple linear progression but rather exhibits a significant inverted-U trajectory. Moderate social crowding can maximize tourists’ social presence and excitement. Yet, consistent with the optimal stimulation level perspective (Mehta, 2013), once the crowd density crosses a specific threshold, the information overload and spatial constraints triggered by excessive congestion begin to erode consumers’ psychological defenses, leading to a rapid decline in positive experiences (Eroglu et al., 2005).

Third, negative emotions suppress impulsive buying rather than inducing compensatory buying, while positive emotions demonstrate a significant positive impact on impulsive buying. Within congested retail settings exemplified by Pang Donglai, tourists’ negative affective states, including agitation and apprehension, predominantly stem from spatial oppression, queuing fatigue, or sensory overload. Therefore, compensatory consumption resulting from threats to self-esteem, diminished perceived control, and a lack of social belonging Mandel et al. (2017) does not materialize in this specific scenario. Concurrently, we tentatively posit that the physical constraints of cross-city travel substantially amplify the conservative nature of non-local consumers’ decision-making under negative emotional states. Unlike local retail contexts where negative emotions might trigger immediate compensatory gratification (Mandel et al., 2017), the unique context of internet-famous supermarkets, which heavily attracts cross-regional visitors, introduces strict logistical boundaries for this specific demographic. Our semi-structured interviews strongly confirm this defensive mechanism. Non-local visitors explicitly cited “the inconvenience of long-distance transportation” and the “fear of spoilage during transit” as primary reasons for severely restricting their purchases, particularly when already exhausted by severe crowding and long queues (Interviewee B and F, see S4 in the Supplementary Materials). Consequently, the synergistic effect of spatial fatigue and practical logistical constraints drives cross-city shoppers toward highly conservative, defensive decision-making. Rather than engaging in compensatory impulsive buying, they prioritize escaping the stressful environment or purchasing only essential, easily transportable items, effectively eliminating unplanned expenditures. Within the internet-famous supermarket setting, positive affect prompts tourists to diminish their cognitive evaluation. Furthermore, to sustain their hedonic shopping experiences, tourists typically rationalize unplanned expenditures as self-gratification or a tangible continuation of their favorable travel encounters, thereby markedly elevating the likelihood of impulsive buying (Karl et al., 2021).

Fourth, perceived spatial crowding negatively affects impulsive buying solely through the mediation of positive emotions, while perceived social crowding does not exert a significant indirect impact on impulsive buying through either emotional pathway. This study revealed that spatial constraints suppress impulsive buying primarily by depriving consumers of positive hedonic experiences, rather than operating through the compensatory mechanisms of negative emotions. Meanwhile, the effect of social crowding on impulsive buying is predominantly driven by direct social contagion rather than being strictly mediated by internal emotional states. This phenomenon is largely attributable to environmental stress, which predisposes tourists toward proactive avoidance strategies instead of compensatory consumption behaviors. Within the hyper-saturated pedestrian traffic of Pang Donglai, the frantic purchasing of trending merchandise readily provokes competitive anxiety among visitors. Consequently, tourists are compelled to modify their shopping itineraries to secure limited commodities, culminating in a deprivation of experiential autonomy. Additionally, once social crowding surpasses a critical threshold, the psychological safety perimeters of tourists are compromised (L. Li et al., 2025), thereby intensifying their adverse psychological appraisals concerning insecurity (Feng et al., 2022), elevated risk (M. Shen et al., 2023), and spatial confinement (Levav & Zhu, 2009).

Fifth, brand trust significantly and negatively moderates the relationships between perceived social crowding and both impulsive buying and positive emotions. As visually demonstrated in the interaction plots (Figure 4 and Figure 5 in Section 4.5), our empirical results challenge the conventional assumption that brand trust universally buffers negative environmental stimuli. Instead, brand trust unexpectedly acts as a restricted, context-dependent boundary condition in internet-famous retail environments. This counter-intuitive dampening effect can be explained through the Expectancy Disconfirmation Theory (Oliver, 1980) and is strongly corroborated by our post hoc qualitative interviews. Consumers with high brand trust (the dashed lines), such as repeat customers or those traveling long distances with specific quality expectations for items like tea or oil, experience severe “expectancy disconfirmation” when the retail environment degrades due to extreme social crowding. Instead of seeking emotional compensation through shopping, their rational cognitive mechanisms are reactivated. As one regular shopper explicitly noted, even when surrounded by frantic buyers, she relies on rational judgment rather than blindly following the crowd (Interviewee A, see S4 in the Supplementary Materials). Similarly, another high-trust consumer emphasized prioritizing actual needs over impulsive urges under crowded conditions (Interviewee B, see S4 in the Supplementary Materials). Conversely, consumers with lower initial brand expectations (the solid lines), often casual tourists simply passing by, demonstrate higher psychological resilience to crowding. Furthermore, they are notably more susceptible to the “herd mentality” and social contagion (J.-G. T. Li et al., 2009). In our interviews, casual visitors admitted that observing others eagerly selecting items directly stimulated their curiosity and drove them to participate in herd consumption (Interviewee C, see S4 in the Supplementary Materials). Some even confessed to spontaneously joining the scramble for unplanned items simply because they were influenced by the surrounding crowd’s behavior (Interviewee D, see S4 in the Supplementary Materials). Thus, in hyper-popular retail settings, high brand trust may inadvertently activate rational restraint, while a lack of rigid expectations paradoxically facilitates crowd-induced impulsive buying. Concerning the non-significant moderating effects on pathways involving spatial crowding, this physiological oppression is exceptionally resilient. Operating as a psychological indicator, brand trust lacks the cross-dimensional capacity to neutralize the direct physical oppression inflicted by spatial constraints.

### 5.2. Theoretical Contribution

First, this study uncovers the emotional differentiation mechanism of perceived crowding in internet-famous supermarket contexts, thereby broadening the applicability frontiers of environmental psychology theory. Conventional environmental psychology predominantly conceptualizes crowding as a unidimensional adverse stimulus, corroborating its positive association with negative emotions (Machleit et al., 1994). While a limited number of investigations have acknowledged the double-edged sword nature of perceived crowding (Byun & Mann, 2011; Zhang et al., 2018), there is a paucity of studies on the specific contexts encompassing both retail and tourism attributes. This study substantiates the divergent effects of the dual facets of perceived crowding, namely perceived physical crowding and perceived social crowding, on bipolar emotions comprising positive and negative emotions within internet-famous supermarkets. This study attempted to enrich the knowledge of environmental stress theory articulated by Stokols (1972). It establishes that in environments characterized by dual retail and tourism properties, perceived social crowding may transmute into a favorable indicator of a vibrant ambiance instead of functioning merely as social duress, consequently introducing context-specific boundary conditions to environmental psychology theories. More importantly, by identifying the inverted-U trajectory of social crowding, this study establishes the specific boundary conditions for this positive signaling effect. This finding empirically echoes the core propositions of environmental stress theory (Stokols, 1972) and retail crowding frameworks (Pons et al., 2006). It demonstrates that while previous literature has widely debated whether crowding yields predominantly positive or negative outcomes, in the specific context of internet-famous retail environments, the hedonic utility of crowd density operates under a strict critical threshold. This bridges the theoretical gap between general environmental stress assumptions and the reality of nonlinear emotional responses in high-density, hyper-popular retail contexts.

Second, this study incorporates brand trust as a moderator, thereby broadening the explanatory frontiers of cognitive appraisal theory within emotional consumption studies. Existing literature predominantly emphasizes the direct influence of brand trust on purchase intention, while its dynamic moderating mechanism linking environmental stress and emotional responses has been largely overlooked in prior studies. Furthermore, this study critically re-evaluates the conventional postulate of cognitive buffering attributed to brand trust, uncovering the intricate effects of brand trust within non-local tourist shopping scenarios. Existing studies generally argue that brand trust mitigates experiences through attributional shift (L. Li et al., 2025; Yao et al., 2013). Nevertheless, this research identifies a divergent operational pathway. Specifically, brand trust negatively moderates the positive associations between perceived social crowding and both impulsive buying and positive emotions. This means that high trust unexpectedly leads to purchase restraint and lessens the positive emotions generated by large crowds. This discovery adds depth to the concept of the universal buffering effect of trust introduced by Doney and Cannon (1997). It confirms that under specific environmental strains such as oversaturated crowds, brand trust not only fails to provide its usual buffering effect but might also increase consumer sensitivity to negative environmental feedback by raising their experience expectations, which consequently magnifies negative experiences to some extent.

### 5.3. Management Implications

This research elucidates the complex interplay among perceived crowding, emotional responses, impulsive buying, and brand trust within the context of internet-famous supermarkets. By synthesizing these empirical insights with the real-world operational practices of Pang Donglai, managers can refine and elevate their management strategies across various critical areas:

First, practitioners must execute differentiated intervention strategies across physical and social environments to systematically mitigate spatial crowding pressure while harnessing the bandwagon effect associated with social crowding. The current research demonstrates that spatial crowding substantially exacerbates negative affective states, whereas social crowding functions as a quality signal that positively elicits positive emotions. Consequently, retailers must avoid conceptualizing crowding as a unidimensional risk and instead adopt differentiated management approaches across physical and social domains. In terms of spatial governance, micro-spatial optimization should prioritize high-frequency retail zones, popular product displays, checkout queues, and tourist convergence points. Given that spatial crowding significantly diminishes positive emotions and amplifies negative emotions, interventions may include proportionally expanding aisle dimensions, optimizing shelf proximity and orientation, and deploying intelligent monitoring systems for the real-time detection of high-density zones. Localized spatial pressure can thus be alleviated via staggered admission, dynamic capacity management, and mobile-based wayfinding notifications. Transitioning to the strategic utilization of social crowding, retailers such as Pang Donglai with established brand equity and public visibility should transcend the mere dispersion of foot traffic and focus on strategically channeling the social proof value embedded within dense crowds. Given that social crowding positively influences positive emotions and impulsive buying, digital displays or informational markers positioned at entrances, high-demand sections, or queuing bottlenecks can broadcast real-time data regarding top-selling items, consumer endorsement metrics, and time-sensitive inventory updates to translate abstract popularity into tangible quality cues. Furthermore, providing peak-hour communication training for frontline staff encourages them to elucidate queuing and crowding dynamics to visitors with greater empathy and transparency to diminish waiting-induced frustration while reinforcing consumer trust in both the merchandise and the establishment. Ultimately, by integrating meticulous physical space management with the directive narrative of the social milieu, enterprises can transmute conventional crowding stress into an immersive consumption potential characterized by both structural order and social dynamism. Furthermore, given the newly identified inverted-U relationship between social crowding and positive emotions, managers should avoid blindly pursuing maximum foot traffic. Instead, efforts should focus on identifying and maintaining the “optimal peak” of crowding. Enterprises should utilize digital technologies, such as heat maps, to activate early warning systems and implement dynamic crowd control before density reaches the critical threshold that deteriorates the experience, ensuring that the bustling atmosphere does not devolve into suffocating oppression for tourists.

Second, high-trust brands should proactively manage tourist expectations to mitigate the expectancy disconfirmation that arises when severe social crowding clashes with elevated brand expectations. The current research reveals that brand trust negatively moderates the relationship between perceived social crowding and positive emotions, indicating that high-trust tourists experience a sharper decline in positive affect when confronted with severe social crowding. For highly trusted brands like Pang Donglai, consumers frequently formulate substantial expectations before even entering the consumption environment. Greater brand trust inherently elevates tourists’ psychological baseline expectations concerning service excellence, spatial order, and the holistic shopping experience. In such contexts, if operational failures such as peak-hour congestion, prolonged queues, inefficient spatial flows, or delayed service responses surpass consumer tolerance thresholds, brand trust can inadvertently heighten tourists’ sensitivity to environmental stressors. This phenomenon occurs due to expectancy disconfirmation, which intensifies negative affective states and dampens positive consumption responses. Therefore, it is imperative for high-trust brands to intensify antecedent expectation management during peak visitation periods. Consistent with the negative moderation finding, brands can leverage official social media channels, video platforms, and in-store announcements to preemptively disseminate holiday traffic projections, peak-time warnings, off-peak shopping suggestions, and auxiliary service plans. Such measures facilitate the development of pragmatic experience expectations among tourists prior to store entry, thereby aligning pre-visit expectations with actual crowding conditions and reducing the expectancy disconfirmation that the moderation effect predicts. Furthermore, allocating mobile service staff to high-density zones during peak hours can provide visitors with navigational assistance and emotional reassurance, which may help alleviate the negative emotional states that the present findings identify as a key deterrent to impulsive buying. Sustaining brand credibility through preemptive communication and superlative care effectively confines potential experience disparities within tolerable limits, ultimately converting latent trust crises into enduring emotional attachments.

Third, retailers should safeguard positive emotional experiences rather than exploit crowding-induced emotional volatility, thereby enhancing customer brand trust and loyalty. The current research demonstrates that positive emotions significantly promote impulsive buying, whereas negative emotions significantly suppress it. This asymmetric pattern indicates that the primary behavioral driver in crowded internet-famous supermarket environments is positive affect, not the compensatory mechanism triggered by negative affect. Nevertheless, when considering long-term brand equity development and customer relationship management, retail organizations ought to refrain from exploiting crowding-induced emotional volatility solely as a promotional lever. They should proactively exercise a value-guiding function to advocate for more rational, measured, and sustainable consumption patterns. Given that the present findings identify positive emotions as the primary pathway to impulsive buying, managers should focus on preserving and enhancing the hedonic experiences that generate positive affect, such as the vibrant social atmosphere and the sense of shared festivity, rather than leveraging negative emotional states for short-term sales gains. Drawing upon the operational ethos of Pang Donglai, which perennially prioritizes genuine service, customer reverence, and rational consumption, businesses can persistently impart the principles of needs-based purchasing, quality prioritization, and the avoidance of overstocking. This can be operationalized through courteous notices, staff advisories, merchandise specifications, and social media broadcasting. Consistent with the finding that negative emotions deter rather than drive impulsive buying, although this strategy ostensibly dampens short-term sales incentives, it practically serves to curtail irrational acquisitions provoked by emotional contagion and conformity psychology within congested atmospheres. This mitigation subsequently alleviates post-purchase regret and bolsters consumer alignment with the core values of the brand. Moreover, the finding that brand trust negatively moderates the social crowding and impulsive buying link suggests that high-trust consumers are less susceptible to social contagion cues. This implies that cultivating brand trust can serve as a natural buffer against herd-driven impulsive consumption. Retailers can reinforce this buffer by consistently delivering on brand promises and maintaining service quality even under crowded conditions, thereby strengthening the internal trust cue that supersedes the external social proof cue. This socially responsible commercial paradigm of this nature effectively subdues the impulsive consumption urges generated by crowded environments. Furthermore, it cultivates steadfast loyalty and organic word-of-mouth promotion that extend beyond basic commercial exchanges, culminating in a multifaceted synergistic outcome of customer satisfaction, brand loyalty, and corporate social responsibility.

### 5.4. Research Limitations and Future Directions

Although this research has revealed the underlying mechanism of how perceived crowding affects purchasing behavior in the context of internet-famous supermarkets, it inevitably contains certain limitations constrained by objective conditions and methodological designs. These limitations warrant further in-depth exploration and expansion in future research:

First, in terms of data structure and measurement methodology, the current research relies heavily on cross-sectional data to conduct a static assessment of tourists’ emotional states. This approach makes it difficult to rigorously establish the dynamic causal relationship between perceived crowding and emotional arousal. Additionally, the cross-sectional design, in which all variables were collected from the same respondents at a single time point, limits the strength of causal inferences and leaves common method bias a concern. Future research could adopt a time-lagged longitudinal design, collecting data on perceived crowding, emotions, and impulsive buying at separate time points, to further mitigate common method bias and strengthen causal claims. Emotional reactions are fundamentally characterized by acute temporal sensitivity and context dependence. Therefore, the psychological experiences of tourists navigating high-density environments are likely to manifest progressive shifts in response to prolonged exposure to crowding or spatial transitions across different shopping settings. To address this gap, future research could employ the Experience Sampling Method to track momentary emotional shifts throughout the shopping journey (Yu et al., 2020). Moreover, the data collection period was extended to comprehensively account for the impact of Pang Donglai’s evolving crowd management policies and operational changes, which may introduce temporal heterogeneity in consumer responses. Although the core measurement instrument remained consistent, this temporal spread introduces potential heterogeneity in tourist perceptions due to seasonal fluctuations and shifting retail environments. Future research should consider adopting a more concentrated data-collection window, incorporating time as a control variable, or conducting multi-group comparisons across survey waves to explicitly test the stability of the observed relationships. Furthermore, by triangulating subjective reports with objective neurophysiological metrics like eye-tracking and Galvanic Skin Response, researchers can precisely delineate emotional peaks at the onset of crowding exposure, pinpoint critical tolerance thresholds, and map the subsequent dynamic adaptation process (S. Li et al., 2023). Additionally, regarding the measurement model, while the CR for all constructs comfortably exceeded the recommended threshold, ensuring strong internal consistency, the AVE values for perceived spatial crowding (0.498) and perceived social crowding (0.460) were marginally below the conventional 0.50 benchmark. Although statistically acceptable given the robust CR values, this highlights a potential limitation in convergent validity. Future research should consider refining these measurement scales to better capture the complex, multifaceted nature of crowding perceptions specifically within hyper-dense internet-famous retail contexts.

Second, the individual heterogeneity of tourists, encompassing personality traits and consumption habits, frequently acts as a pivotal moderator linking emotional arousal to ultimate purchasing decisions. Nevertheless, due to the specific scope of the current investigation, these elements were temporarily excluded from the analytical framework. Subsequent studies are encouraged to integrate micro-level individual variables such as the Big Five personality model (particularly extraversion and neuroticism) (Qureshi et al., 2025), alongside trait impulsive buying tendencies and hedonic versus utilitarian shopping motivations (Y. Wang et al., 2020). Exploring these factors will help unpack their distinct moderating effects on the pathway in which perceived crowding dictates purchasing behavior through emotional responses. For instance, investigating whether high sensation-seeking individuals or hedonic consumers exhibit heightened sensitivity to the popularity effects generated by social crowding would significantly elucidate the individual boundary conditions governing this mechanism.

Third, the scope of the current investigation is largely restricted to the native Chinese context, without fully isolating the underlying effects of macro-level factors like relational consumption philosophies and high-density social settings on the empirical outcomes. Consequently, this constraint partially limits the cross-situational generalizability of the conclusions. Given that consumers from different cultural backgrounds exhibit significant differences in defining physical space boundaries and relying on social cues, subsequent research could adopt a cross-cultural perspective. This approach would help further clarify the differentiated transmission pathways through which perceived crowding triggers impulse purchasing within globalized, multicultural internet-famous supermarkets, ultimately bolstering the theoretical model’s explanatory capacity and global relevance.

## Figures and Tables

**Table 1:** Demographic information of participants.

	N	Percentage (%)		N	Percentage (%)
**Gender**			**Occupation**		
Male	277	49	Civil servant	34	6
Female	288	51	Managerial staff	48	8.5
**Age**			Professional/technical staff	62	11
18 to 24	245	43.4	Service and sales personnel	35	6.2
25 to 35	171	30.3	Blue-collar worker	31	5.5
36 to 45	108	19.1	Student	192	34
46 to 60	37	6.5	Production operator	8	1.4
61 or Order	4	0.7	Self-employed	58	10.3
**Education**			Retired	5	0.9
High school and below	78	13.8	Other	92	16.3
Junior college	118	20.9	**Monthly Income**		
Bachelor’s degree	328	58.1	2000 and below	171	30.3
Postgraduate and above	41	7.3	2001 to 4000	101	17.9
**Place of Residence**			4001 to 6000	135	23.9
Xinxiang City proper	0	0	6001 to 8000	64	11.3
Other counties in Xinxiang City	59	10.4	8001 to 10,000	45	8
Other cities in Henan Province	284	50.3	above 10,000	49	8.7
Other provinces or regions	222	39.3			

**Table 2:** Confirmatory factor analysis results.

Constructs and Scale Items	Standard Loading	Cronbach’s α	AVE	CR
**Perceived spatial crowding**		0.818	0.498	0.793
1. The supermarket does not appear spacious.	0.559			
2. The supermarket does not give a sense of openness.	0.566			
3. I feel constrained while shopping in this supermarket.	0.851			
4. I experience a sense of restriction during the shopping process.	0.796			
**Perceived social crowding**		0.799	0.460	0.766
1. In my view, the supermarket is very crowded.	0.815			
2. The supermarket is somewhat overly busy due to the large number of people.	0.791			
3. There are many customers in the supermarket.	0.518			
4. The flow of people in the supermarket is high during the shopping process.	0.533			
**Positive emotions**		0.888	0.668	0.889
1. This shopping experience made me excited.	0.776			
2. This shopping experience made me happy.	0.846			
3. This shopping experience made me satisfied.	0.856			
4. This shopping experience made me pleasantly surprised.	0.787			
**Negative emotions**		0.921	0.707	0.923
1. I regret coming to Pang Donglai for shopping.	0.819			
2. This shopping experience made me feel disappointed.	0.873			
3. This shopping experience made me feel upset.	0.894			
4. This shopping experience made me feel annoyed.	0.849			
5. This shopping experience made me feel irritable.	0.763			
**Brand trust**		0.916	0.651	0.918
1. I believe this supermarket is trustworthy.	0.753			
2. I believe this supermarket is reliable.	0.848			
3. I believe that purchasing products at this supermarket is safe and secure.	0.860			
4. I feel safe and reassured when buying products at this supermarket.	0.862			
5. I believe this supermarket is very honest.	0.801			
6. If any issues occur with a product, I believe this supermarket will handle related problems effectively.	0.704			
**Impulsive buying**		0.760	0.543	0.777
1. I bought more items than I had originally planned.	0.573			
2. While shopping in the supermarket, I purchased some items I had not initially intended to buy.	0.788			
3. While shopping in the supermarket, I came across some products that were unplanned but that I wanted to purchase.	0.824			

Note: Goodness-of-fit: χ^2^/dƒ = 2.845, RMSEA = 0.057, IFI = 0.942, TLI = 0.933, CFI = 0.942.

**Table 3:** Results of the discriminant validity test.

Constructs	1	2	3	4	5	6
1. Perceived spatial crowding	0.706					
2. Perceived social crowding	0.603	0.678				
3. Positive emotions	−0.345	−0.173	0.817			
4. Negative emotions	0.553	0.294	−0.510	0.841		
5. Brand trust	−0.125	0.208	0.251	−0.381	0.807	
6. Impulsive buying	−0.063	0.082	0.352	−0.237	0.379	0.737

Note: The values on the diagonal represent the square root of the average variance extracted, and the values in the lower triangle are the correlation coefficients between the corresponding variables.

**Table 4:** Heterotrait–monotrait ratio (HTMT) results.

Constructs	1	2	3	4	5	6
1. Perceived spatial crowding						
2. Perceived social crowding	0.459					
3. Positive emotions	0.370	0.184				
4. Negative emotions	0.590	0.226	0.515			
5. Brand trust	0.189	0.290	0.252	0.396		
6. Impulsive buying	0.133	0.102	0.390	0.271	0.410	

**Table 5:** Descriptive statistics of each dimension and results of the normality test for items.

Constructs	Item	Mean	SD	Skewness	Kurtosis	Population Mean	Population SD
Perceived spatial crowding	SPA1	2.860	1.032	0.036	−0.456	2.874	0.845
	SPA2	2.980	1.057	−0.053	−0.692		
	SPA3	2.930	1.057	0.005	−0.686		
	SPA4	2.720	1.054	0.342	−0.451		
Perceived social crowding	SOC1	3.640	1.007	−0.598	0.070	4.000	0.702
	SOC2	3.790	0.976	−0.778	0.387		
	SOC3	4.300	0.771	−1.438	3.249		
	SOC4	4.280	0.774	−1.269	2.561		
Positive emotions	PE1	3.390	0.756	−0.077	0.634	3.468	0.672
	PE2	3.510	0.759	−0.283	0.705		
	PE3	3.560	0.764	−0.273	0.605		
	PE4	3.400	0.825	−0.127	0.395		
Negative emotions	NE1	2.160	0.821	0.573	0.481	2.188	0.727
	NE2	2.210	0.827	0.477	0.196		
	NE3	2.150	0.812	0.570	0.643		
	NE4	2.110	0.788	0.499	0.361		
	NE5	2.310	0.919	0.466	−0.145		
Brand trust	BT1	4.130	0.718	−0.923	2.153	4.220	0.583
	BT2	4.190	0.706	−1.047	2.875		
	BT3	4.270	0.697	−1.122	2.932		
	BT4	4.260	0.685	−0.916	2.101		
	BT5	4.230	0.684	−0.859	2.022		
	BT6	4.240	0.674	−0.813	1.746		
Impulsive buying	IB1	3.730	0.878	−0.320	−0.335	3.749	0.679
	IB2	3.700	0.817	−0.653	0.479		
	IB3	3.820	0.778	−0.875	1.289		

**Table 6:** Structural model evaluation indices and hypothesis test outcomes.

Hypotheses No.	Hypothesized Relationships	Standard Path Loadings	Standard Error	Critical Ratio	*p*-Value	Hypothesis Test Outcome
H1a	Perceived spatial crowding → Impulsive buying	0.039	0.057	0.405	0.686	Rejected
H1b	Perceived social crowding → Impulsive buying	0.161 ^a^	0.091	2.157	0.031	Supported
H2a	Perceived spatial crowding → Negative emotions	0.675 ^b^	0.056	9.599	0.000	Supported
H2b	Perceived spatial crowding → Positive emotions	−0.491 ^b^	0.049	−7.110	0.000	Supported
H2c	Perceived social crowding → Negative emotions	−0.134 ^a^	0.101	−2.169	0.030	Supported
H2d	Perceived social crowding → Positive emotions	0.138 ^a^	0.098	2.071	0.038	Supported
H3a	Negative emotions → Impulsive buying	−0.157 ^a^	0.048	−2.431	0.015	Rejected
H3b	Positive emotions → Impulsive buying	0.321 ^b^	0.050	5.402	0.000	Supported

Note: ^a^ statistically significant (*p* < 0.05); ^b^ statistically significant (*p* < 0.001).

**Table 7:** Results of the moderating effect test.

Path	Path Coefficients	Standard Error	*t*-Value	*p*-Value	Results
H5a: Perceived spatial crowding × Brand trust → Impulsive buying	−0.064	0.054	−1.185	0.237	Rejected
H5b: Perceived social crowding × Brand trust → Impulsive buying	−0.105 ^a^	0.044	−2.363	0.018	Rejected
H6a: Perceived spatial crowding × Brand trust → Negative emotions	0.027	0.051	0.524	0.600	Rejected
H6b: Perceived spatial crowding × Brand trust → Positive emotions	−0.057	0.054	−1.066	0.287	Rejected
H6c: Perceived social crowding × Brand trust → Negative emotions	−0.022	0.046	−0.494	0.621	Rejected
H6d: Perceived social crowding × Brand trust → Positive emotions	−0.103 ^a^	0.046	−2.258	0.024	Rejected
H7a: Negative emotions × Brand trust → Impulsive buying	−0.059	0.053	−1.101	0.271	Rejected
H7b: Positive emotions × Brand trust → Impulsive buying	−0.051	0.048	−1.049	0.294	Rejected

Note: ^a^ statistically significant (*p* < 0.05).

**Table 8:** Results of the index of moderated mediation effect tests.

Path	Moderating Variable Level	Effect Value	Standard Error	95% Confidence Interval
H8a: Perceived spatial crowding × Brand trust → Negative emotions → Impulsive buying	Low brand trust (M − 1 SD)	−0.034	0.025	[−0.086, 0.014]
	Hight brand trust (M + 1 SD)	−0.056	0.030	[−0.115, 0.003]
	Difference value (Δ)	−0.022	0.037	[−0.095, 0.053]
H8b: Perceived spatial crowding × Brand trust → Positive emotions→ Impulsive buying	Low brand trust (M − 1 SD)	−0.054	0.018	[−0.093, −0.021]
	Hight brand trust (M + 1 SD)	−0.057	0.022	[−0.106, −0.022]
	Difference value (Δ)	−0.003	0.026	[−0.057, 0.044]
H8c: Perceived social crowding × Brand trust → Negative emotions → Impulsive buying	Low brand trust (M − 1 SD)	−0.035	0.021	[−0.082, −0.003]
	Hight brand trust (M + 1 SD)	−0.041	0.025	[−0.096, −0.001]
	Difference value (Δ)	−0.006	0.029	[−0.064, 0.051]
H8d: Perceived social crowding × Brand trust → Positive emotions → Impulsive buying	Low brand trust (M − 1 SD)	−0.022	0.016	[−0.061, 0.003]
	Hight brand trust (M + 1 SD)	−0.048	0.028	[−0.110, −0.005]
	Difference value (Δ)	−0.025	0.027	[−0.076, 0.032]

## Data Availability

The data presented in this study are available upon request from the corresponding author. The data are not publicly available due to confidentiality and ethics reasons.

## Note

1 Source: https://www.henan.gov.cn/2024/10-11/3072225.html (accessed on 8 October 2024).

## Supplementary Materials

The following supporting information can be downloaded at: https://www.mdpi.com/article/10.3390/bs16091710/s1.
