# Supplementary material for: How Perceived Crowding Shapes Tourists’ Impulsive Buying in Internet-Famous Supermarkets: The Roles of Bipolar Emotions and Brand Trust
Source: Behav Sci (Basel). 2026 Sep 21;16(9):1710. doi: 10.3390/bs16091710 (PMC13603945; doi:10.3390/bs16091710)
Supplement: Supplementary file 1 [file behavsci-16-01710-s001.zip › behavsci-4466010-supplementary.pdf]

Supplementary Materials

S1. Semi-structured interviews

To provide in-depth contextual explanations for statistical findings, especially unexpected or nonsignificant ones, thereby achieving triangulation of quantitative breadth and qualitative depth that enhances both the credibility and interpretive power of the conclusions, semi-structured interviews were conducted from December 20, 2024 to January 13, 2025. Semi-structured, face-to-face interviews were carried out on-site at Pang Donglai (Xinxiang stores) during peak weekend hours. Respondents were randomly intercepted in high-density areas (e.g., checkout lines, popular product displays). Each interview lasted approximately 10 to 15 minutes. All qualitative data were recorded, transcribed, and analyzed using thematic analysis to extract behavioral motivations specific to the internet-famous supermarket context.

S2. Demographic Profile of Interviewees

While multiple interviews were conducted, Table A1 details the profiles of the core interviewees whose insights were most representative of the collective behavioral patterns observed and were utilized to inform the discussion section of this study.

Table S1. Profile of the core qualitative interviewees

Interviewee ID (in Text)	Gender	Age	Origin (Distance)	Travel Mode	Primary Motivation	Brand Trust Level
A	Female	62	Anyang, Henan Province (Cross-city)	High-speed rail	Targeted specific purchase	High (Repeated loyalist)
B	Male	40	Hebei Provinces (Cross-province)	Self-driving	Tourism & shopping	High (Pre-planned expectation)
C	Male	31	Yuanyang, Henan Province (Cross-county)	Self-driving	Casual shopping & checking-in	Low/Moderate
D	Male	38	Pingdingshan, Henan Province (Cross-city)	Self-driving	Casual shopping & checking-in	Low/Moderate
E	Male	20	Xinyang, Henan Province (Cross-city)	Self-driving	Tourism & checking-in	Moderate
F	Female	36	Shandong Province (Cross-province)	High-speed rail	Tourism & checking-in	High (Brand reputation)
G	Male	40	Shandong Province (Cross-province)	Self-driving	Tourism & specific purchase	High (Rational expectation)
H	Male	22	Luoyang, Henan province (Cross-city)	Self-driving	Tourism & checking-in	Moderate

S3. Semi-Structured Interview Protocol

The interview guide was developed based on the Stimulus-Organism-Response (SOR) framework and contextualized for internet-famous retail environments. The core guiding questions were categorized into four thematic modules:

Theme 1: Contextual Boundary and Motivation

Q1: Is your primary purpose for visiting Pang Donglai today purely for shopping, or is it combined with tourism and experiential “checking-in”?

Q2: Compared to routine shopping at supermarkets in your hometown, how does your mindset and physical itinerary differ here?

Theme 2: Crowding Perception and Tolerance Thresholds

Q3: At what level of human density do you perceive the crowd as a positive signal of “popularity and vibrancy”?

Q4: At what specific critical threshold does this vibrancy transition into a sense of physical oppression or psychological threat?

Theme 3: Emotional Responses and Compensatory Consumption

Q5: When environmental crowding triggers anxiety or fatigue, do you engage in unplanned purchasing (compensatory consumption) to alleviate these negative emotions? If not, why?

Q6: How do logistical constraints (e.g., long-distance travel, carrying capacity) shape your purchasing decisions when you feel environmentally stressed?

Theme 4: Brand Trust and Expectancy Disconfirmation

Q7: Did you hold exceptionally high expectations regarding the service and environment prior to your arrival?

Q8: When the severe physical crowding clashes with your high pre-visit expectations, does this psychological discrepancy (expectancy disconfirmation) alter your trust and subsequent purchasing behavior?

S4. Representative Verbatim Quotes Supporting Core Findings

The following translated excerpts from the raw interview transcripts provide the qualitative evidence underpinning the theoretical arguments in Section 5.1 (Conclusion and Discussion).

(1) Evidence for the Insignificant Effect of Spatial Crowding on Impulsive Buying (Target-oriented Rationality & Buffering):

“Inside, they leave enough space for you to step and move. The overall venue is quite spacious, and picking items is relatively convenient... The store's spatial layout and signage are very clear. If I want to find something, I just look up at the signs and locate it quickly.” — Interviewee E (Male, 20)

“I won't blindly follow the crowd or buy impulsively just because it's crowded. I will actively ask others if the product is actually good. Even if many people are fighting for it, I won't be led astray... I stick to my planned list.” — Interviewee A (Female, 62)

“I came here with a set target: to buy beer, and I don't plan to buy anything else. A large crowd only indirectly suggests that the product has a good reputation, but it doesn't mean it is something I need. I focus solely on selecting what I need and don't pay attention to the crowds around me.” — Interviewee H (Male, 22)

(2) Evidence for Negative Emotions Suppressing Compensatory Consumption (Logistical Constraints):

"Because we live very far away in Shandong, we took the high-speed rail. For heavy items like cooking oil or large boxes of detergent, it is fundamentally inconvenient to carry them back on the train. So, even if I feel frustrated by not getting the beer I wanted, I definitely won't just buy a bunch of other heavy things to compensate." — Interviewee F (Female, 36)

"When it gets too crowded, you just feel exhausted. Many items on our planned list were given up because we couldn't squeeze in or they were out of stock. We prioritize actual practical needs and wouldn't buy randomly just because we are anxious." — Interviewee B (Male, 40)

(3) Evidence for the Negative Moderating Role of Brand Trust (Expectancy Disconfirmation vs. Herd Mentality):

(High Trust / Restraint): "We are 40 years old, with kids, and we travel here specifically with needs. We don't like to consume impulsively... Even if I see everyone rushing to buy something, I won't buy it unless it's useful to us. We have enough life experience to remain rational, regardless of how highly the brand is praised." — Interviewee G (Male, 40)

(Low/Moderate Trust / Conformity): "I didn't plan to buy much, but when you are in this environment, it's hard not to be influenced. I saw everyone grabbing oatmeal, so I also grabbed two packs. It's the herd mentality; seeing others scramble for it makes you curious to try." — Interviewee D (Male, 38)

(Low/Moderate Trust / Conformity): "When there are so many people, seeing others picking items triggers my curiosity... It is definitely a conformity consumption psychology. Even if I don't know the product well, seeing others buy it makes me want to get one too." — Interviewee C (Male, 31)
